# Objective Multi-Metric Fusion for Critical Node Identification via CRITIC and Global–Local Context Modeling

**DOI:** 10.3390/e28080927

**Published:** 2026-08-18

**Authors:** Pengcheng Cai, Canjv Lu, Ying Huang, Yi Xie

**Affiliations:** College of Electronic Engineering of NUDT, Hefei 230037, China; caipengcheng93@163.com (P.C.); lucanju17@nudt.edu.cn (C.L.); wind1134@163.com (Y.H.)

**Keywords:** complex networks, critical node identification, graph attention network, multi-metric fusion

## Abstract

Accurately identifying critical nodes in complex networks and applying targeted protection strategies significantly enhances network security. Traditional importance metrics rely on a single topological feature and cannot fully capture node influence. Existing multi-attribute fusion methods integrate multiple structural sources but typically use fixed weights or predefined rules, failing to adaptively adjust attribute contributions based on local and global network characteristics, which limits their generalization across diverse networks. To address this, we propose the CRITIC-based Objective Weighting and Multi-Metric Fusion Method (COWMF). COWMF first builds a Graph Attention Network with Virtual Global–Local Integration (GAT-VGL), taking four low-complexity topological metrics, degree centrality (DC), H-index, degree and neighborhood information centrality (DNC), and k-shell, as input. Through a learnable attention mechanism, GAT-VGL adaptively aggregates multi-hop neighborhood information and explicitly incorporates global structural information via a virtual node to achieve whole-graph topological awareness, generating a global influence score with good discriminative power and high computational efficiency. This score is then integrated with DC and DNC into an improved CRITIC-based objective weighting fusion scheme, enabling adaptive synergy among local connectivity, semi-local radiation, and global structure. Experiments on six real-world networks of varying types and scales show that COWMF demonstrates relatively stable and competitive performance in both simulated attack and susceptible-infected-recovered (SIR) spreading simulations, two complementary experiments, demonstrating satisfactory disruptive capability and propagation influence. Its importance scores exhibit high monotonicity across all networks, with good discriminative power.

## 1. Introduction

Complex networks have emerged as an effective modeling framework for characterizing the intrinsic relational structures of complex systems and have been widely applied to the analysis and understanding of diverse real-world systems [1]. Numerous systems, such as the hierarchical topology of the Internet, the path connectivity of urban transportation networks [2], and the interaction patterns among users in social media, can be formally represented as graph models composed of nodes and edges [3,4], where nodes denote entities and edges represent various types of relationships among them [5]. In recent years, research on complex networks has attracted considerable attention in both fundamental scientific exploration and engineering applications [6]. Critical node identification is one of the core research directions in this field, as a small number of highly influential nodes often play a decisive role in the overall functional stability and information propagation efficiency [7,8,9]. For instance, identifying critical components in road transportation networks improves traffic resilience and mitigates congestion under disruptions, and recognizing key nodes in power grids is crucial for preventing cascading failures and ensuring stable operation [10,11]. Therefore, identifying influential nodes in complex networks holds not only theoretical significance but also substantial practical value [12,13]. Given its central role in complex network research, this problem continues to receive extensive academic interest [14,15]. Existing studies indicate that network topology and its fundamental characteristics play a decisive role in the identification of influential nodes [16,17].

Early metrics for quantifying node importance can be broadly divided into two categories, namely local metrics and global metrics. The former use only information from a node’s immediate neighborhood (i.e., direct neighbors), exemplified by degree (DC) [18] and H-index [19]. Although these metrics efficiently reflect local connectivity activity, they fail to capture a node’s global structural role. The latter evaluate the influence of individual nodes from a global topological perspective by analyzing data across all nodes, such as betweenness centrality (BC) [20] and k-shell [21]. These global metrics effectively identify critical bridge nodes or core-layer members but often overlook subtle differences in local connectivity, resulting in insufficient resolution within structurally similar regions. To balance local detail and global structure, researchers have further proposed semi-local metrics, such as the work by Han et al. [22], which incorporates information from both direct neighbors and partially extended neighborhoods. However, any single metric captures only one aspect of network topology. Consequently, recent studies have attempted to enhance overall performance through hybrid frameworks that fuse multi-source information. Nevertheless, existing multi-metric fusion methods still suffer from significant limitations. On one hand, many approaches adopt fixed weights or predefined rules, leading to weak generalization and unstable performance across diverse networks. On the other hand, some methods incorporate either low-discrimination metrics such as k-shell, which cannot differentiate nodes within the same shell, or computationally expensive metrics such as BC, whose high time complexity renders it impractical for large-scale networks. These issues constrain the robustness and applicability of current fusion methods in real-world complex networks and motivate the development of a more balanced, efficient, and high-resolution framework for influential node identification.

This paper proposes a multi-metric fusion framework for critical node identification. The framework first constructs a Graph Attention Network with Virtual Global–Local Integration (GAT-VGL), which takes four low-complexity topological metrics as input, namely DC, H-index, degree and neighborhood information centrality (DNC), and k-shell. Through a learnable attention mechanism, GAT-VGL adaptively aggregates multi-hop neighborhood information and explicitly incorporates global structural information via a virtual node to achieve whole-graph topological awareness, thereby generating a high-resolution, computationally efficient global influence score. Subsequently, this learned score is integrated with DC and DNC using the CRITIC-based Objective Weighting and Multi-Metric Fusion Method (COWMF), which jointly characterizes node importance from three complementary perspectives: local connectivity strength, semi-local radiation capacity, and data-driven global propagation potential. The COWMF procedure consists of four steps. First, DC, DNC, and the GAT-VGL score are extracted from the target network. Second, range normalization is applied to eliminate scale differences among the three metrics. Third, the Criteria Importance Through Intercriteria Correlation (CRITIC) method [23] is employed to compute fully data-driven objective weights by combining the normalized Shannon entropy of each metric with inter-metric conflict. Finally, a composite importance score is obtained through weighted summation, enabling high-precision node ranking.

The main contributions of this work are summarized as follows.

(1) To address the high computational cost of traditional global metrics such as BC and the low discriminative power of metrics such as k-shell, we design GAT-VGL. This model takes only low-complexity topological metrics as input and learns a global influence score that achieves both global awareness and improved discriminative resolution. The learned score can be generated with high computational efficiency, significantly enhancing scalability on large-scale networks.

(2) We abandon subjective weighting or simple averaging strategies and instead adopt CRITIC. By combining the normalized Shannon entropy of each metric with inter-metric conflict, CRITIC dynamically computes fully data-driven objective weights. This enables the fusion process to adaptively reflect the intrinsic topological structure of the network, substantially improving the objectivity and stability of node evaluation.

(3) The proposed COWMF generally achieves favorable node ranking performance across six diverse real-world networks. In two complementary validation settings, namely Network Attack Simulation Experiment and information propagation based on the Susceptible–Infected–Recovered (SIR) model, COWMF ranks among the competitive methods. Moreover, the entire pipeline requires no human intervention or task-specific parameter tuning. The method combines interpretability, strong generalization capability, and high computational efficiency, making it well suited for critical node identification in large-scale complex networks.

The remainder of this paper is organized as follows. Section 2 systematically elaborates on and analyzes classical and recently proposed methods for influential node identification. Section 3 details the design rationale and algorithmic workflow of the COWMF method. Section 4 validates the effectiveness of the proposed approach through case studies on real-world networks. Section 5 summarizes the paper and highlights the key contributions and implications of the work.

## 2. Related Work

In the field of complex network analysis, identifying key spreaders is an important task with extensive applications [24]. Numerous studies have been dedicated to developing centrality measurement methods that can accurately identify these critical nodes. This section reviews the latest advances in this field, with a focus on elaborating the contributions and limitations of existing methods. Existing influential node identification methods are generally classified into three categories, namely local, semi-local and global, based on the range of topological information they rely on.

### 2.1. Local-Structure-Based Methods

Local metrics evaluate nodes using only limited information from the nodes itself and its immediate neighbors. These methods are computationally efficient and highly scalable, making them suitable for rapid screening in large-scale networks. Typical examples include DC [18], which reflects a node’s local activity through its direct connection count; the H-index [19], inspired by bibliometric principles, measuring how deeply a node is embedded among high-degree neighbors; and the clustering coefficient, which characterizes the density of triangle closures within the neighborhood. Although these methods offer strong computational efficiency, their discriminative power in complex scenarios is often limited due to the neglect of global network topology.

### 2.2. Global-Structure-Based Methods

Global metrics assess node importance by analyzing the entire network structure, revealing a node’s functional position within the macroscopic topology. Representative methods include BC, closeness centrality (CC), k-shell decomposition, and EC. BC [20] precisely captures a node’s role as a communication hub by counting its occurrences across all shortest paths. CC [25] reflects the average distance from a node to all others, indicating its information dissemination efficiency. k-shell [26] reveals core–periphery structures through iterative peeling, offering high computational efficiency but lacking fine-grained differentiation within shells. To address this limitation, researchers have proposed several local extensions based on k-shell, such as ELKSS [27], which constructs local scores by aggregating k-shell values from two-hop neighborhoods. However, these methods overemphasize the k-shell contributions of neighbors while downplaying the node’s intrinsic structural properties, leading to potential misclassifications in structurally heterogeneous networks. EC [28] models long-term influence by iteratively aggregating neighbor importance, yet it tends to be dominated by highly connected nodes and its convergence depends heavily on network connectivity. Overall, although global metrics provide strong interpretability at the macro level, they generally lack sensitivity to local connection patterns and struggle to fully capture a node’s structural characteristics at the neighborhood scale.

### 2.3. Semi-Local-Structure-Based Methods

To balance local detail with global perspective, semi-local metrics incorporate multi-hop neighborhood information beyond immediate neighbors. For instance, Yi et al. [29] proposed LASPN, a semi-local importance measure grounded in the concept of extended neighborhoods. The core idea integrates a node’s own local influence, the semi-local influence of nearby nodes, and the relative change in local average shortest path based on extended neighborhoods, further highlighting the distinctive features of semi-local metrics. Zhao et al. [30] summarized DNC, a semi-local metric that synergistically combines a node’s degree, the sum of its neighbors’ degrees, and neighborhood link quality, effectively integrating local activity with the influence of near neighbors. Recently, Fu et al. [31] proposed MNNGE, a fusion method based on multilayer neighbor gravity and information entropy. This method first constructs initial node weights by combining degree and k-shell values, then quantifies the interaction between nodes and their neighborhoods through relative and direct gravitational forces, where the latter leverages common neighbor structures, and adaptively integrates the entropy centrality of first- and second-order neighbors using information entropy. Ultimately, it provides a fine-grained characterization of key roles within local expansion structures.

### 2.4. Multi-Attribute Fusion Methods

Single importance metrics typically reflect only one aspect of a node’s role across the entire network, making it difficult to comprehensively assess its influence in complex networks from multiple perspectives. To address this limitation, numerous researchers have explored multi-attribute decision-making approaches. These methods treat various importance metrics as evaluation attributes and generate comprehensive scores via rational weighting and fusion mechanisms, resulting in more holistic assessments. In recent years, several well-established multi-attribute decision models have been introduced into this field. For instance, Wang et al. [32] proposed HKEN, a multi-attribute fusion method for identifying critical nodes. This approach integrates the k-shell value reflecting global position, degree indicating local connectivity strength, local clustering coefficient capturing local cohesion, and Jaccard coefficient measuring structural similarity. By extending influence spreading to second-order neighborhoods, it constructs a comprehensive evaluation metric that simultaneously considers both local and global topological features. Chiranjeevi et al. [33] introduced ISBC, a multi-attribute fusion ranking method that synergistically combines isolating centrality and BC, two complementary structural properties, to significantly improve the accuracy and efficiency of identifying critical nodes in complex networks while accounting for both local clustering effects and global path control. Esfandiari [34] proposed another multi-attribute fusion method addressing the low resolution and core-group issues inherent in k-shell decomposition. By incorporating the k-shell index, the ratio of degree to k-shell value reflecting relative activity within shells, and the ratio of the sum of neighbors’ degrees to the square of the k-shell value indicating local radiation capacity, this method generates normalized weighted scores, substantially enhancing sorting resolution and accuracy while maintaining linear time complexity. Although these methods improve assessment comprehensiveness to some extent, their fusion mechanisms still exhibit notable shortcomings.

### 2.5. Research Gaps and Motivation

As evidenced by the above review, existing influential node identification methods still exhibit significant limitations in capturing multi-scale structural characteristics. Local metrics such as DC and H-index effectively reflect a node’s neighborhood activity but completely overlook its role in the macroscopic network structure. Global metrics can reveal a node’s bridging function or core status, yet they are often insensitive to local connectivity patterns and suffer from issues such as high computational cost or limited fine-grained discriminative power within structurally homogeneous regions. Semi-local metrics achieve a certain balance between local detail and global perspective by incorporating multi-hop neighborhood information, but their evaluation scope is typically constrained by fixed hop counts or predefined decay rules. Although multi-metric fusion strategies have shown promise in integrating heterogeneous topological information, current approaches still face two critical limitations. On one hand, weight assignment commonly relies on manually preset rules or empirical heuristics, lacking the ability to adaptively learn from the intrinsic topological properties of the network, which restricts generalization performance across heterogeneous networks. On the other hand, fusion frameworks often incorporate two types of inefficient metrics: one type includes intrinsically low-discrimination metrics such as k-shell, which causes a large number of nodes to receive highly similar importance scores and severely degrades ranking resolution, the other type comprises computationally expensive global metrics such as BC, whose high time complexity renders them impractical for large-scale networks.

To address these challenges, this paper proposes a two-stage learning and multi-metric fusion framework for critical node identification. The framework first constructs GAT-VGL, taking low-complexity topological metrics, namely DC, H-index, DNC, and k-shell, as input. Through a learnable attention mechanism, GAT-VGL adaptively aggregates multi-hop neighborhood information to characterize local structural influence, while simultaneously leveraging a virtual node to explicitly incorporate global structural information and achieve whole-graph topological awareness, thereby generating a global influence score that achieves both high discriminative power and high computational efficiency. This learned score is then integrated with DC and DNC within COWMF, enabling adaptive synergy across local, semi-local, and global perspectives. The final output is a high-precision, high-resolution, and scalable ranking of critical nodes.

## 3. Proposed Algorithm

Many real-world systems can be abstracted as complex networks composed of nodes and edges, such as social groups, power grids, and transportation networks, where entities within the system are treated as nodes and their interactions or relationships as edges. This paper represents a complex network as an undirected graph G=(V,E), where V={1,2,3,…,n} is the node set and E={1,2,3,…,m} is the edge set [26].

Multi-metric fusion methods can effectively integrate the measurement results of individual structural metrics. When selecting sub-metrics, it is essential to ensure both information complementarity and perspective diversity. To this end, this paper selects representative metrics from three structural perspectives: local, semi-local, and global topology, forming the basis for multi-attribute decision making. The local perspective employs DC, and the semi-local perspective employs DNC. Both are classical structural metrics that can be efficiently computed. The global perspective requires a metric capable of reflecting a node’s macroscopic propagation capacity, but traditional global metrics often suffer from high computational cost or low discriminative power. To address this limitation, this paper proposes the Graph Attention Network [35,36] with Virtual Global–Local Integration (GAT-VGL) to learn and generate a global influence score that is computationally efficient and highly discriminative. This metric, together with DC and DNC, serves as input to the COWMF, ultimately yielding a high-precision, high-resolution, and scalable ranking of critical nodes.

### 3.1. Input Feature Construction for GAT-VGL

To enhance GAT-VGL’s ability to perceive node importance from multiple structural perspectives, its initial node features are composed of four complementary structural metrics: DC and H-index measure local activity, DNC reflects semi-local influence, and k-shell characterizes global coreness. Collectively, these metrics provide structural information at local, semi-local, and global levels as model input.

#### 3.1.1. Degree Centrality

Degree centrality (DC) is a classical structural metric for characterizing the local importance of nodes and is commonly used for critical node identification. Its core idea is that the more direct neighbors a node has, the higher its value. For an undirected network G=(V,E), the degree centrality of node *i* is defined as:

(1) $$DC_{i}=\frac{\deg_{i}}{N-1}$$

where $\deg_{i}$ denotes the degree of node *i*, the number of connections it has to other nodes, and *N* is the total number of nodes in the network. DC is computationally efficient and intuitively interpretable, making it suitable for rapid identification of highly connected nodes.

#### 3.1.2. H-Index

The H-index was originally developed for academic evaluation and later introduced into complex network analysis to assess the connection quality of a node’s neighbors. In complex networks, the H-index of node *i* is defined as the largest integer *h* such that the node has at least *h* neighbors, each with degree no less than *h*. Let $d_{1}\geq d_{2}\geq \ldots \geq d_{k}$ be the degree sequence of node *i*’s neighbors sorted in descending order. The mathematical definition of the H-index is:

(2) $$H_{i}=\max\left\{h:d_{h}\geq h\right\}$$

where $H_{i}$ denotes the H-index of node *i*, $d_{h}$ is the *h*-th value in the descendingly sorted neighbor degree sequence, and *h* is the maximum integer satisfying the condition. This metric considers not only the node’s direct connectivity but also incorporates the influence of its neighboring nodes.

#### 3.1.3. Degree and Neighborhood Information Centrality

In complex networks, DNC characterizes a node’s semi-local influence by aggregating the degrees of nodes within three hops, weighted by an inverse-square distance penalty. It should be noted that the DNC used in this work is an improved version that deviates from its original definition. For an undirected network G=(V,E), the DNC of node i is defined as:

(3) $${\mathrm{DNC}}_{i}=\sum_{j\in V,\;0<d(i,j)\leq 3}\frac{\deg(j)}{d{\left(i,j\right)}^{2}}$$

where $deg_{j}$ denotes the degree of node j, d(i,j) represents the shortest path length between nodes i and j, and the summation spans all other nodes within a maximum distance of three hops. The inverse-square distance decay ensures that closer neighbors contribute more significantly to the node’s local influence. While maintaining computational efficiency, DNC overcomes the limitation of traditional degree centrality, which relies solely on one-hop neighborhoods, enabling it to identify key nodes that may have low degrees but are deeply embedded within densely connected local clusters. This makes DNC well-suited for fine-grained assessment of local structural importance in large-scale networks.

#### 3.1.4. k-Shell

k-shell is a network decomposition method based on recursive removal, used to characterize the nesting depth of nodes within a network. The computation starts from nodes with the smallest degree and iteratively removes all nodes whose current degree is less than or equal to *k*, dynamically updating the degrees of the remaining nodes. After each removal round, all removed nodes are assigned a coreness value of *k*, forming the k-shell. The process then increments *k* and repeats until all nodes in the network have been assigned to a shell.

### 3.2. GAT-VGL Model Architecture

**Figure 1:**
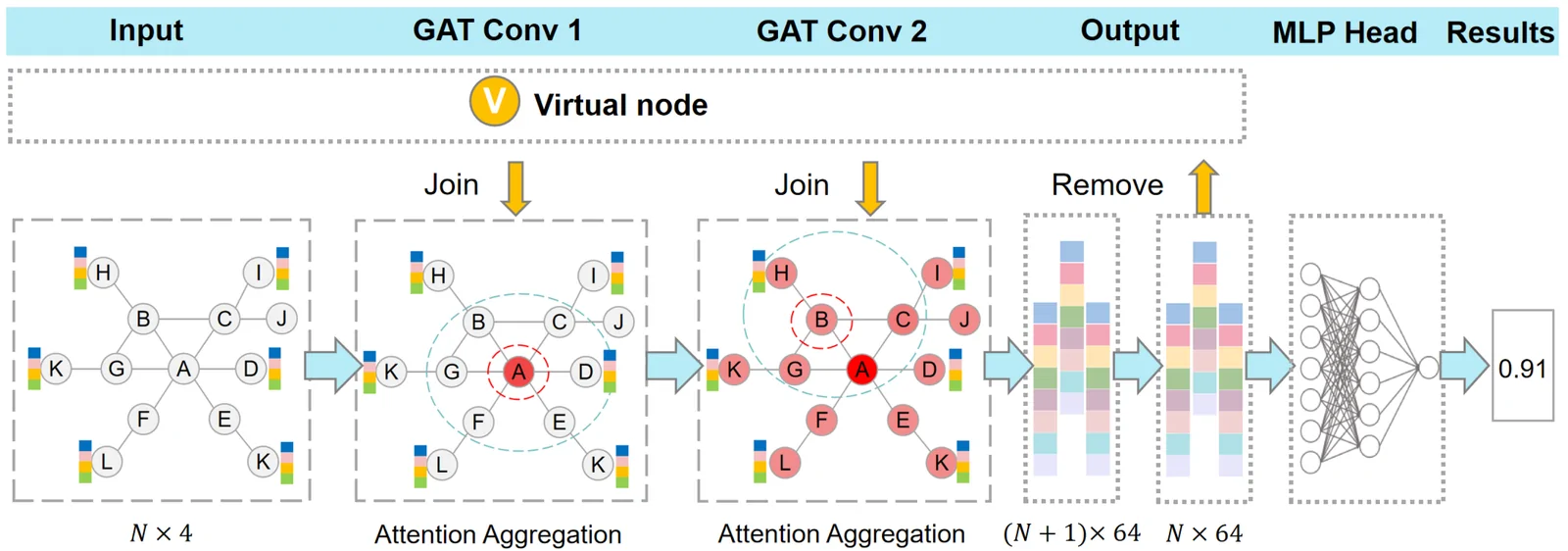
Architecture of the GAT-VGL model with a virtual node for global importance scoring. The model employs a two-layer Graph Attention Network (GAT) augmented with a learnable virtual node connected to all real nodes. Node features are updated via attention-based message passing, followed by non-linear transformations. Final node representations are passed through an MLP head to produce importance scores reflecting each node’s influence in the network.

The architecture of the GAT-VGL model is illustrated in Figure 1. It consists of two stacked Graph Attention Network (GAT) layers [37,38] and introduces a learnable virtual node connected to all real nodes in the graph. This mechanism enables bidirectional interaction between local neighborhood information and global graph-level information, thereby better capturing the overall topological characteristics that are critical for propagation influence.

Let $h_{i}^{\left(l\right)}$ denote the embedding of node *i* at layer *l*, where the initial input corresponds to l=0. The initial feature vector $h_{i}^{\left(0\right)}\in R^{4}$ for each node is composed of four precomputed structural metrics: degree centrality (DC), H-index, degree and neighborhood information centrality (DNC), and k-shell. By integrating multi-scale structural information, the model obtains a more comprehensive and discriminative initial node representation, which lays the foundation for subsequent graph-structure-aware feature aggregation.

The GAT-VGL model stacks L=2 message-passing layers. At each layer, first-order neighbor information is aggregated through the graph attention mechanism. Specifically, a multi-head attention mechanism with 4 heads is employed, with a hidden dimension of 64 per layer and the ELU activation function [39,40]. This process follows the standard GAT computation paradigm, allowing the model to adaptively learn the importance weights of different neighbors and achieve fine-grained information fusion.

Subsequently, the model fuses global topological information through the virtual node mechanism. A learnable virtual node is explicitly added when constructing the graph data object and is connected to all real nodes. The initial embedding of the virtual node is a trainable parameter and is updated synchronously with other nodes during message passing. Through this structural design, information flows bidirectionally between local neighborhoods and the global summary, enabling effective integration of local and global features without requiring additional graph pooling operations.

After L=2 propagation layers, the final representation $h_{i}^{\left(2\right)}$ of each real node is fed into a two-layer MLP decoder with Dropout (dropout rate 0.3) to output a score:

(4) $$s_{i}=\mathrm{MLP}\left(h_{i}^{\left(2\right)}\right).$$

**Figure 2:**
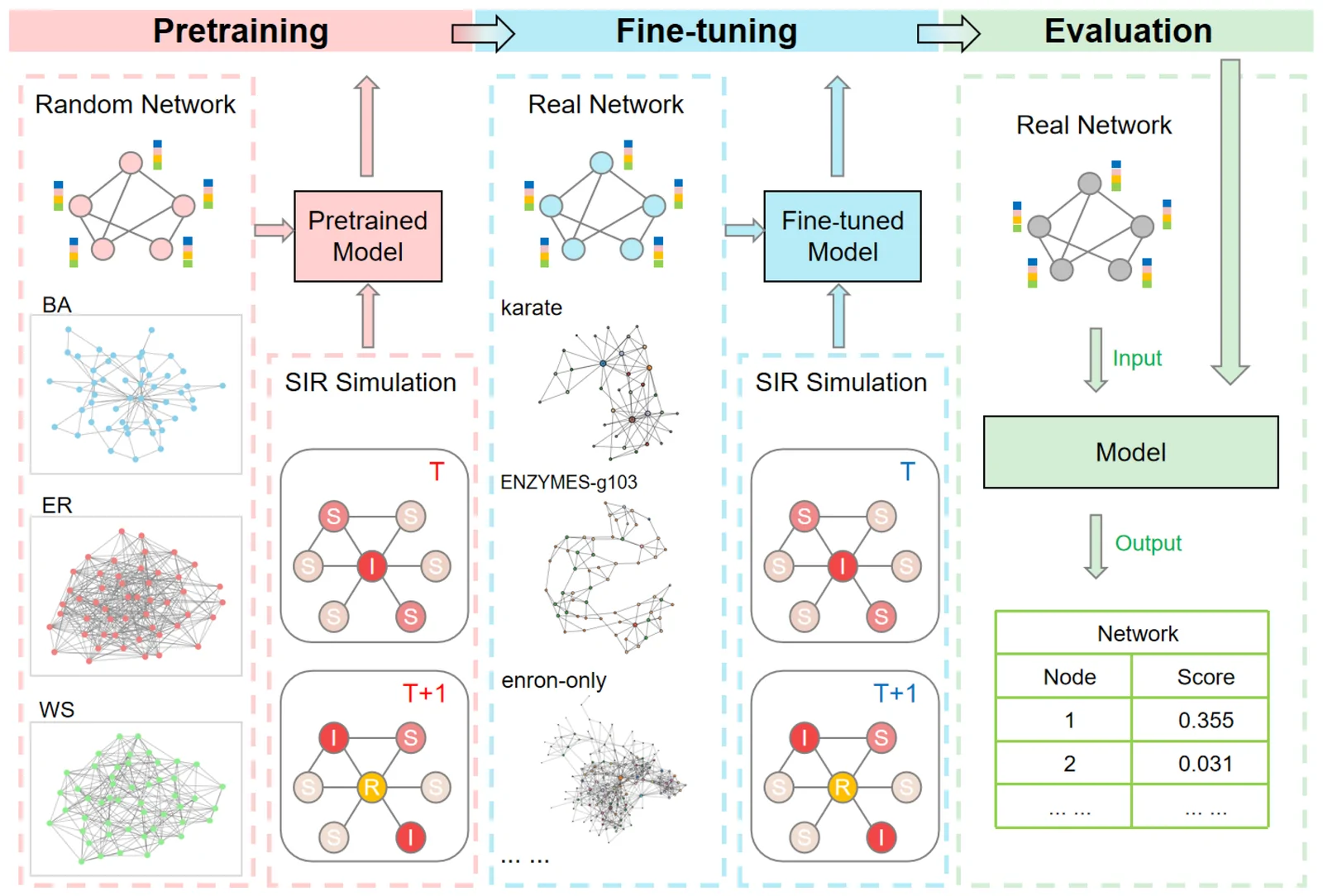
Two-stage training and evaluation pipeline of the GAT-VGL model. In Stage 1, the model is pretrained on a large set of synthetic graphs (BA, WS, ER) using SIR-derived ranking labels with a pairwise ranking loss. In Stage 2, the pretrained model is fine-tuned on a small set of real-world networks to adapt to realistic topological structures. After fine-tuning, the model outputs a global influence score $S_{i}^{\text{GAT-VGL}}$ for each node in the target network.

GAT-VGL is trained in two stages. Since real-world networks are difficult to obtain in large quantities while synthetic networks can be generated indefinitely, we adopt a two-stage training strategy: first learning the ranking patterns of node influence on a large set of synthetic networks, and then fine-tuning on a small number of real-world networks, as shown in Figure 2.

The first stage is pretraining on synthetic graphs. We generate diverse synthetic networks using three classical random graph models: Barabási–Albert (BA), Watts–Strogatz (WS), and Erdős–Rényi (ER). Each model generates 1000 graphs, resulting in a total of 3000 training graphs. The generation parameters for these networks are listed in Table A1 in the Appendix A. The number of nodes in each graph is uniformly sampled between 50 and 250 to cover networks of varying scales.

For each synthetic graph *G*, we first compute its average degree 〈k〉 and second moment of degree $\langle k^{2}\rangle$, and then determine the effective transmission rate for the Susceptible–Infected–Recovered (SIR) model as:

(5) $$\beta =\max\left(0.1,\frac{\langle k\rangle }{\langle k^{2}\rangle -\langle k\rangle }\right),$$

with the recovery rate fixed at γ=0.5. For each non-isolated node as the initial infected seed, we independently run 100 SIR simulation trials and take the average outbreak size as the ground-truth label for that node.

The training objective is not to regress ground-truth label but to learn its ranking order. To this end, we design an efficient pairwise ranking loss that compares nodes in the top-*k* and bottom-*k* positions of the ground-truth rankings. The parameter *k* is adaptively determined based on the number of nodes *n* (i.e., k=30 when n≥60, and k=⌊n/2⌋ otherwise). For networks with a larger number of nodes, fixing k=30 effectively reduces the computational cost and accelerates convergence. Conversely, for smaller graphs, setting k=⌊n/2⌋ ensures a sufficient number of nodes participate in the training process, avoiding the issue of insufficient training samples. We explicitly acknowledge that with this adaptive rule, the selected sets are not always restricted to extreme minorities, but rather cover a substantial portion of the graph when *n* is small. The loss function is defined as:

(6) $$L_{\mathrm{rank}}=\frac{1}{\left|T|\cdot |B\right|}\sum_{i\in T}\sum_{j\in B}\max\left(0,\;0.1-(s_{i}-s_{j})\right),$$

where *T* and *B* denote the sets of top-*k* and bottom-*k* nodes sorted by ground-truth labels, respectively. To ensure training stability and disjoint sets (T∩B=∅), *k* is determined adaptively based on *n*. This adaptive strategy guarantees that the top and bottom sets remain disjoint for every training graph, while maintaining training efficiency across networks of varying sizes. This loss function aims to improve ranking performance, evaluated by the Spearman correlation coefficient and Kendall tau coefficient, while avoiding the high computational cost of full pairwise comparisons.

The model and the StandardScaler are jointly trained for 30 epochs on the 3000 synthetic graphs.

In the second stage, we fine-tune the pretrained model on real-world networks. Although synthetic networks are easy to generate in large numbers, their topological properties differ significantly from those of real-world networks and lack practical representativeness. Therefore, we select 35 small-scale real-world networks, ensuring no overlap with the test set. The detailed list of these networks is provided in Table A3 in Appendix A.

For each real-world network, we compute β using the same formula and generate node labels via SIR simulations. We then load the pretrained GAT-VGL weights and the StandardScaler, and fine-tune the model using the same ranking loss for 20 epochs. To mitigate catastrophic forgetting, the learning rate is set to one-tenth of that used in pretraining, i.e., $1\times 10^{-4}$.

The GAT-VGL score, denoted as $S_{i}^{\mathrm{GAT}-\mathrm{VGL}}$, can be calculated for networks of any scale. Benefiting from the sparse message-passing mechanism of GAT, the inference of GAT-VGL is highly efficient and scales well with network size, allowing for efficient execution even on large-scale sparse networks. This score serves as a high-level, data-driven metric reflecting global influence, which is subsequently used in the COWMF fusion process.

### 3.3. CRITIC-Based Objective Weighting and Multi-Metric Fusion Method

**Figure 3:**
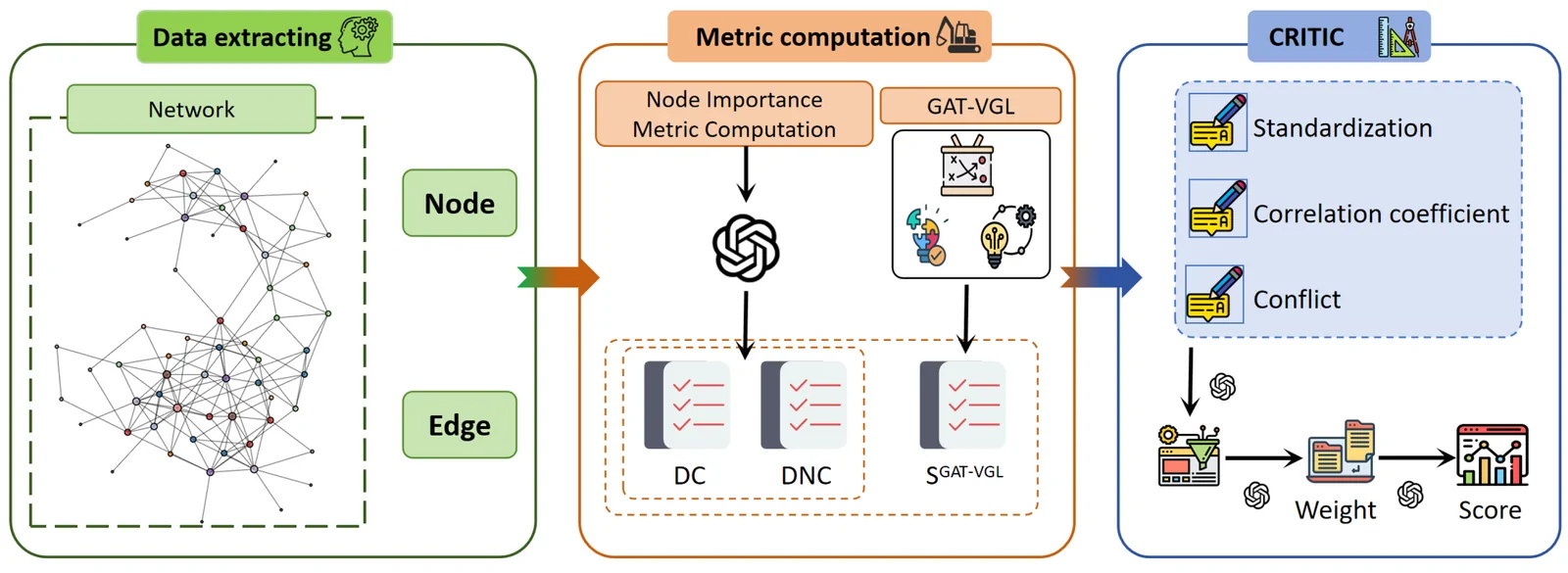
Pipeline of the COWMF method for critical node identification, comprising data extraction, multi-metric computation (DC, DNC, and GAT-VGL), and CRITIC-based objective weighting.

To comprehensively evaluate node importance and avoid the bias of any single metric, this paper adopts a multi-metric fusion strategy that integrates local structural information, semi-local structural information, and global topological information to construct a composite node importance metric, COWMF. The computational pipeline is illustrated in Figure 3. Specifically, we select DC as a representative of local connectivity strength, DNC to capture a node’s local influence within a three-hop neighborhood, and GAT-VGL to reflect global topological information, forming the following base metric set:

$$M=\{\mathrm{DC},\mathrm{DNC},S^{\mathrm{GAT}-\mathrm{VGL}}\}.$$

It is worth noting that all three metrics underlying COWMF can be computed with high computational efficiency. The computation of DC is extremely fast with negligible cost. DNC is implemented via breadth-first search limited to three hops, and its graph-wide computation remains highly efficient on sparse networks. Although the GAT-VGL score is model-generated, its inference relies solely on four low-complexity topological features as input. Among them, the H-index for each node *i* is computed with low overhead, which incurs negligible cost on real-world sparse networks; k-shell decomposition is performed once via a global peeling algorithm and the results are reused across all nodes. Thanks to the sparse message-passing mechanism of GAT, the overall inference complexity of GAT-VGL scales with high computational efficiency as network size increases.

Therefore, despite integrating perspectives from local, semi-local, and global levels, COWMF incurs significantly lower computational overhead than traditional high-complexity global metrics such as betweenness centrality, demonstrating strong scalability and suitability for identifying critical nodes in large-scale networks.

To eliminate dimensional differences among metrics, range normalization is first applied to their original values. Let there be n nodes, and let $x_{ij}$ denote the original value of the *i*-th node on the *j*-th metric, Define $x_{j}^{\min}=\min_{1\leq k\leq n}x_{kj}$ and $x_{j}^{\max}=\max_{1\leq k\leq n}x_{kj}$. The normalization formula is:

(7) $$z_{ij}=\frac{x_{ij}-x_{j}^{\min}}{x_{j}^{\max}-x_{j}^{\min}}$$

where $x_{j}^{\min}$ and $x_{j}^{\max}$ denote the minimum and maximum values of the *j*-th metric across all nodes in the entire network, respectively. The normalized result is denoted as $z_{ij}\in \left[0,1\right]$, which represents the standardized score of the *i*-th node on the *j*-th metric.

On this basis, we introduce an improved CRITIC method for objective weighting. Compared with the original CRITIC method that typically employs standard deviation to measure metric dispersion, this paper adopts an equal-frequency normalized Shannon entropy to quantify the dispersion of each metric, thereby enhancing robustness against non-Gaussian distributions. The improved method holds that the information content of a metric is jointly determined by its internal variation and its conflict with other metrics: the greater the variation and the weaker the correlation with others, the more independent information it provides, and thus the higher its weight should be.

Specifically, the Pearson correlation coefficient between each pair of normalized metrics is first computed. Let ${\bar{z}}_{j}=\frac{1}{n}\sum_{i=1}^{n}z_{ij}$ denote the mean value of the *j*-th normalized metric across all network nodes. Then the Pearson correlation coefficient between any two metrics *j* and *k* is defined as:

(8) $$r_{jk}=\frac{\sum_{i=1}^{n}\left(z_{ij}-{\bar{z}}_{j}\right)\left(z_{ik}-{\bar{z}}_{k}\right)}{\sqrt{\sum_{i=1}^{n}{\left(z_{ij}-{\bar{z}}_{j}\right)}^{2}}\cdot \sqrt{\sum_{i=1}^{n}{\left(z_{ik}-{\bar{z}}_{k}\right)}^{2}}}$$

Here, ${\bar{z}}_{j}$ and ${\bar{z}}_{k}$ denote the means of the normalized *j*-th and *k*-th metrics, respectively, and $r_{jk}$ measures their linear correlation, with values in [−1,1].

According to the core idea of the CRITIC method, the information contribution of each metric is jointly determined by its own variation and its conflict with other metrics. Since network metrics often exhibit non-Gaussian distributions, this paper employs quantile-based normalized Shannon entropy to quantify the dispersion of each metric:

(9) $$H_{j}=\frac{-\sum_{b=1}^{B}p_{jb}\log_{2}p_{jb}}{\log_{2}B}$$

In this context, *B* denotes the number of quantile bins, and we set B=30 in this paper; $p_{jb}$ represents the probability that the *j*-th metric falls into the *b*-th quantile interval, and $H_{j}\in \left[0,1\right]$ is the normalized Shannon entropy. A larger value of $H_{j}$ indicates a higher discriminability of this metric among nodes.

The conflict of the *j*-th metric is defined as follows:

(10) $${\text{Conflict}}_{j}=\sum_{k=1,\;k\neq j}^{3}\left(1-r_{jk}\right)$$

where $r_{jk}$ denotes the Pearson correlation coefficient between the *j*-th and the *k*-th metrics. The value of ${\text{Conflict}}_{j}$ reflects the information independence of the *j*-th metric. Specifically, when $r_{jk}$ approaches 1, the metrics are highly positively correlated, indicating strong redundancy and a minimal conflict contribution. When $r_{jk}$ approaches −1, the metrics exhibit a strong negative correlation, representing a significant directional conflict and yielding a larger conflict value. Furthermore, when $r_{jk}$ approaches 0, the metrics are nearly linearly independent, providing distinct information. Therefore, a larger value of ${\text{Conflict}}_{j}$ corresponds to stronger information independence.

Combining the above two components, the raw information content of the *j*-th metric is obtained as:

(11) $$C_{j}=H_{j}\cdot {\text{Conflict}}_{j}$$

where $C_{j}$ is the comprehensive information content of the *j*-th metric. Finally, objective weights are derived by normalizing the information content across all metrics:

(12) $$w_{j}=\frac{C_{j}}{\sum_{k=1}^{3}C_{k}},\;j=1,2,3$$

where $w_{j}$ is the objective weight of the *j*-th metric.

Based on these weights, the normalized metric values are aggregated via weighted summation to yield the proposed composite node importance metric, COWMF:

(13) $${\mathrm{COWMF}}_{i}=w_{1}\cdot z_{i1}+w_{2}\cdot z_{i2}+w_{3}\cdot z_{i3}$$

A larger COWMF value indicates higher comprehensive importance of node i. This metric is entirely based on a data-driven objective weighting mechanism, effectively fuses local, semi-local, and global topological features, requires no human intervention or post-processing enhancement, and thus possesses strong interpretability and generalization capability.

### 3.4. Computational Complexity Analysis

To evaluate the scalability of the proposed COWMF framework, we analyze the time complexity of each component. Let *N* denote the number of nodes and *M* denote the number of edges.

The calculation of DC and k-shell decomposition both rely on traversing the graph structure, with a time complexity of O(N+M). The H-index calculation involves sorting the degree sequence of neighbors for each node. Its complexity is $O(\sum_{i=1}^{N}k_{i}\log k_{i})$, where $k_{i}$ is the degree of node *i*. In sparse networks where M∝N, this is approximately O(Nlog〈k〉). The DNC metric requires a bounded Breadth-First Search (BFS) up to 3 hops for each node. The complexity is $O(N\cdot {\bar{b}}^{3})$, where $\bar{b}$ is the average branching factor. While more computationally intensive than DC, it remains linear with respect to the number of edges in sparse networks, i.e., O(M), and is significantly more efficient than global metrics.

In the design of COWMF, we aim to integrate metrics that capture network properties from different scales: DC for local connectivity, DNC for semi-local radiation, and a global metric for global propagation potential. While Betweenness Centrality (BC) is a classic choice for capturing global structure and offers high discriminative power, its computational cost is prohibitive for networks beyond a few thousand nodes. Therefore, we employ the GAT-VGL model to learn and generate a global influence score.

The standard calculation for BC such as Brandes’ algorithm has a complexity of O(NM) for unweighted graphs. In contrast, the total time complexity of our GAT-VGL based approach consists of two main stages: feature preparation and model inference.

- Feature Preparation: This stage involves computing the input metrics for the GAT-VGL model.
  - DC: The complexity is O(M), as it requires iterating through all edges to count node degrees.
  - k-shell: The complexity is O(N+M), as it iteratively removes the lowest-degree nodes and their incident edges until no nodes remain, with each node and each edge processed at most once.
  - H-index: The complexity is $O(\sum_{i=1}^{N}k_{i}\log k_{i})$, which is approximately O(Nlog〈k〉) in sparse networks.
  - DNC: This requires a bounded BFS for each node, with a complexity of $O(N\cdot {\bar{b}}^{3})$, where $\bar{b}$ is the average branching factor.
- Model Inference: The GAT-VGL inference itself involves a fixed number of Graph Attention layers L=2 and feature dimensions F=4. Its complexity is dominated by the message passing mechanism, calculated as O(L·M·F). Since *L* and *F* are small constants, the inference time scales linearly with the number of edges, i.e., O(M).

In practice, this combined process is substantially faster than calculating BC, making GAT-VGL highly efficient for networks with thousands to tens of thousands of nodes.

To empirically validate this efficiency, we generated synthetic networks (BA, WS, ER) with 5000 and 10,000 nodes and measured the wall-clock time for GAT-VGL inference (including feature computation) and exact BC calculation. The results, presented in Table 1, demonstrate that our approach is more efficient than BC, confirming its high efficiency for networks with thousands to tens of thousands of nodes.

## 4. Data and Experiments

To evaluate the performance of COWMF, we selected six real-world network datasets with diverse scales and topological characteristics for experimentation. These networks are bn-cat-mixed-species_brain_1 (denoted as BrainCat hereafter), sandi-auths, retweet, Anaheim_traffic, SC-TS, and US-Grid. The proposed COWMF method is designed for undirected and unweighted graphs. When encountering directed or weighted networks, we ignore the edge directions and weights, and remove duplicate edges by retaining only one copy for each pair of nodes connected by multiple edges. Furthermore, all isolated nodes are removed from the datasets. The specific structural properties and data sources of each network are presented in Table 2. In this table, V denotes the total number of nodes, E denotes the number of edges, Dmax represents the maximum degree among all nodes, and KSmax indicates the highest k-shell value across all nodes.

The comparison includes five mainstream centrality metrics, DC, H-index, DNC, BC, and k-shell, and three recently proposed metrics, LASPN, HKEN, and ISBC. Among these, DC, BC, and k-shell are computed directly using built-in functions from the NetworkX library, while H-index is implemented programmatically according to its original definition. The DNC metric is employed here in an improved form that modifies its original formulation. LASPN, HKEN, and ISBC are reproduced strictly following the algorithmic procedures and technical details described in their respective original publications. All experiments are conducted on a laptop equipped with an AMD Ryzen 9 5900HS processor and 32 GB of memory, using Python 3.11 for implementation and NetworkX 3.6.1 for graph manipulation and fundamental network analysis.

### 4.1. Comprehensive Performance Evaluation in Multi-Network Scenarios

To comprehensively evaluate the generalization capability of the COWMF metric across diverse networks, this study conducts systematic experiments on six real-world networks with significant structural differences. These include brain networks, collaboration networks, retweet networks, transportation road networks, biological networks, and power networks. These networks exhibit distinct characteristics in terms of size, sparsity, and topological structure, thereby enabling a rigorous assessment of the robustness and adaptability of key node identification methods under heterogeneous network topologies.

#### 4.1.1. SIR Spreading Simulation

To evaluate the influence of different node importance ranking algorithms on spreading dynamics, this paper employs the classical Susceptible–Infected–Recovered (SIR) model to perform spreading simulation experiments on the selected networks [41,42]. In the SIR model, each node is assigned one of three states: susceptible, infected, or recovered. Initially, a single node is randomly selected as the seed node and set to the infected state, while all other nodes are in the susceptible state. At each time step, an infected node transmits the infection to its susceptible neighbors with infection probability β, and simultaneously transitions to the recovered state with recovery probability γ, after which it no longer participates in the spreading process.

Under the aforementioned parameter settings, multiple node importance metrics were employed to rank all nodes in each network. The top 5% of nodes according to each ranking were selected as initial seeds to launch independent SIR spreading simulations. The cumulative number of infected nodes over time was recorded to quantitatively assess the spreading efficiency of different seeding strategies, with results averaged over 100 independent simulation runs.

The infection rate β used in this study is defined as:

(14) $$\beta =\max\left(0.1,\;\frac{\langle k\rangle }{\langle k^{2}\rangle -\langle k\rangle }\right)$$

where 〈k〉 denotes the average degree of the network and $\langle k^{2}\rangle$ represents the second moment of the degree distribution. This formulation of β balances spreading speed with intrinsic network structural properties, preventing premature termination of spreading due to excessive sparsity while ensuring sufficient dynamical richness. The recovery rate is fixed at 0.5 to allow infected nodes a reasonable window of opportunity to propagate the infection before recovery, thereby enhancing the persistence and observability of the spreading process and avoiding overly rapid convergence.

**Figure 4:**
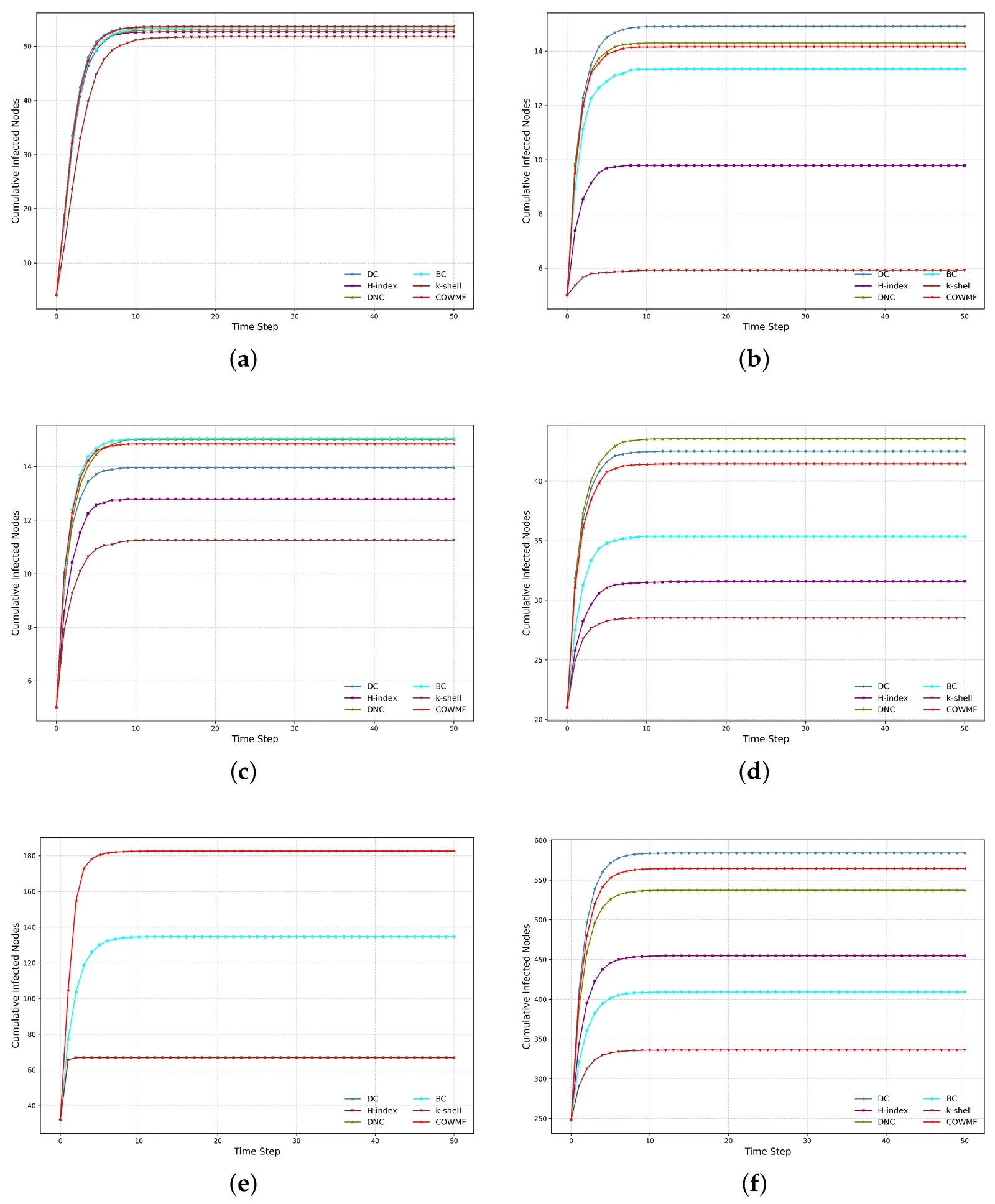
Comparison of SIR spreading dynamics when top-ranked nodes identified by the COWMF importance indicator and classical importance metrics are used as initial infection seeds across six real-world networks. (**a**) BrainCat; (**b**) sandi-auths; (**c**) retweet; (**d**) Anaheim_traffic; (**e**) SC-TS; (**f**) US-Grid. The horizontal axis denotes the time step, and the vertical axis shows the average cumulative number of infected nodes over 100 independent simulations.

**Figure 5:**
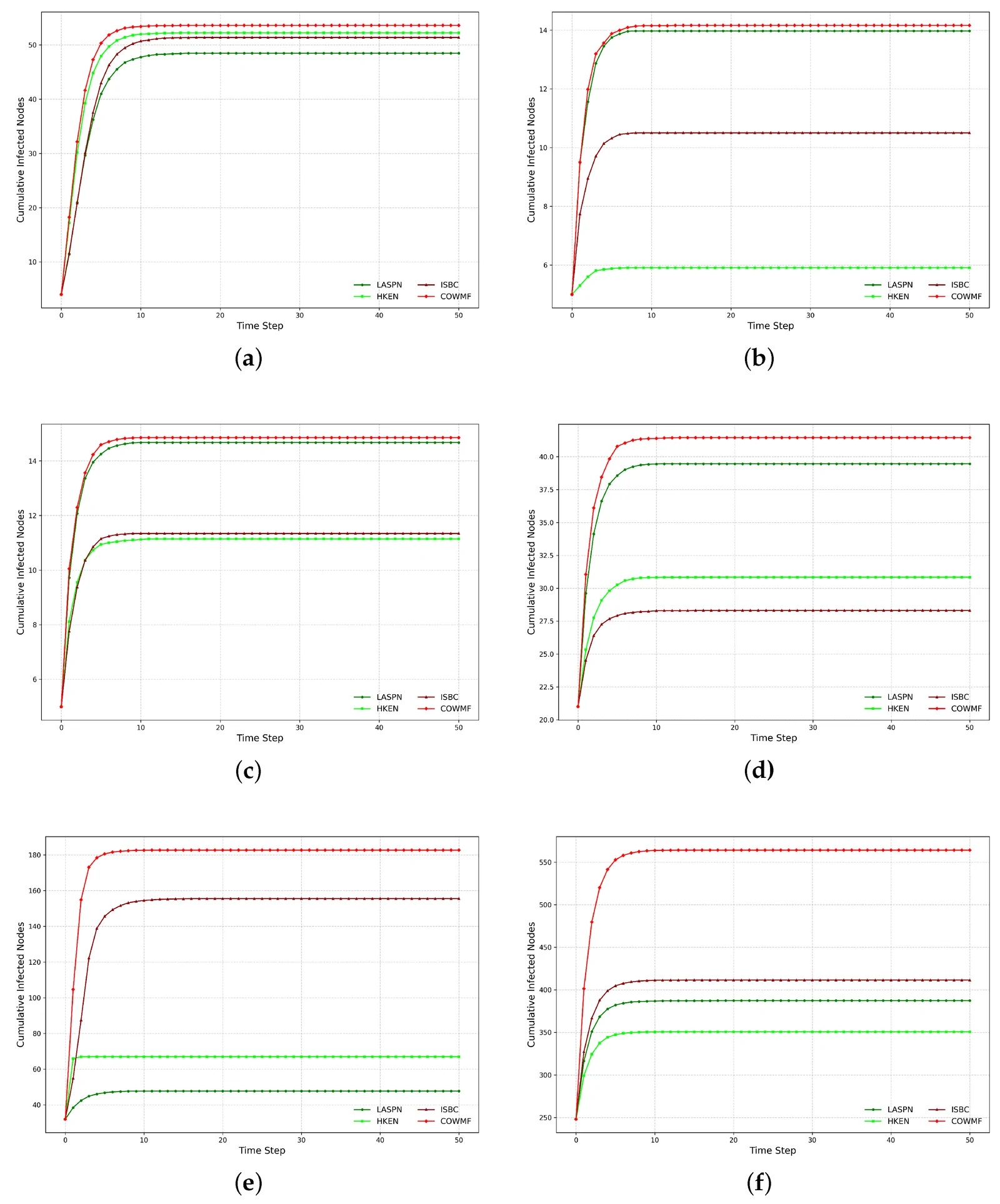
Comparison of SIR spreading dynamics when top-ranked nodes identified by the COWMF importance indicator and other recently proposed importance metrics are used as initial infection seeds across six real-world networks. (**a**) BrainCat; (**b**) sandi-auths; (**c**) retweet; (**d**) Anaheim_traffic; (**e**) SC-TS; (**f**) US-Grid. The horizontal axis denotes the time step, and the vertical axis shows the average cumulative number of infected nodes over 100 independent simulations.

Under the above parameter settings, multiple node importance metrics are employed to rank all nodes in each network. The top 5% of nodes according to each ranking are selected as initial seeds to launch independent SIR spreading simulations. The cumulative number of infected nodes over time is recorded to quantitatively assess the spreading efficiency under different seeding strategies. The results, averaged over 100 independent simulation runs, are presented in Figure 4 and Figure 5, which display the temporal evolution of the cumulative infection ratio across the six real-world networks. The horizontal axis represents the time steps of the spreading process, and the vertical axis denotes the cumulative number of infected nodes.

Figure 4 presents a comparison between COWMF and classical node importance metrics, including DC, H-index, DNC, BC, and k-shell, while Figure 5 compares COWMF with recently proposed node importance metrics, namely LASPN, HKEN, and ISBC. To provide a more quantitative comparison, we calculated the area under the curve (AUC) for each method across the six networks, as shown in Table 3 and Table 4. The detailed rankings and the average rank are presented in Table 5. In the table, Avg Rank denotes the average rank, and Rank is determined based on the average rank, where a smaller average rank indicates a higher position. In cases of identical average ranks, the final ordering is determined by the standard deviation of the ranks across networks; a smaller standard deviation indicates higher stability and results in a higher ranking. When calculating the individual rankings within each network, ties are allowed.

As shown in the figures and table, COWMF achieves the favorable average rank (1.83) and demonstrates relatively stable performance. It ranks first in the BrainCat, retweet, and SC-TS networks, and maintains a top-four position in the other networks, ranking fourth in sandi-auths and second in US-Grid and Anaheim_traffic. In contrast, the performance of other methods shows some fluctuations across different networks. For instance, DC ranks first in sandi-auths and US-Grid but ranks eighth in SC-TS. Similarly, ISBC performs well in SC-TS but shows lower performance in BrainCat, retweet, and Anaheim_traffic.

Furthermore, to verify the robustness of our method under different spreading conditions, we conducted extensive experiments with varying infection rates (β). Table 6, Table 7, Table 8, Table 9, Table 10 and Table 11 present the results for β=0.05,0.2,0.5, including both the final infected size and the AUC. Across these different settings, COWMF consistently demonstrates excellent performance, confirming its effectiveness and stability as a node importance metric.

In summary, the performance of existing methods tends to vary across different network architectures, indicating a lack of consistent robustness. In contrast, COWMF demonstrates comparable SIR spreading efficiency and maintains a relatively good level of performance across diverse real-world networks, suggesting its effectiveness and broad applicability as a general-purpose node importance metric.

#### 4.1.2. Simulated Attack Experiment

In the simulated attack experiment of node removal on networks, there are generally two classical methods: single-node attack experiment and successive node attack experiment [43]. This study focuses on undirected and unweighted networks. For datasets that are originally directed or weighted, we convert them into undirected and unweighted forms by ignoring edge directions and weights. Furthermore, the initial networks are not required to be fully connected and may naturally consist of multiple connected components.

In the single-node attack experiment, the network is reset to its original state after removing one node each time, which ensures that all subsequent attacks are conducted independently on the initial network topology. This method can only reflect the instantaneous perturbation caused by single-node failure to the network structure, and cannot characterize the system evolution process under the coordinated failure of multiple nodes.

In contrast, the successive node attack experiment does not restore the network after each removal. It sorts nodes in descending order according to their importance scores, and removes nodes sequentially based on the sorting result. This process simulates the gradual degradation of network structure under sustained and cumulative attacks, which is more consistent with actual scenarios. This method not only effectively avoids the random deviation caused by independent evaluation in single-node attacks, but also can identify the key node sequence that has the most significant impact on network robustness, and can quantify the critical point of network functional collapse, such as the inflection point where the scale of the Largest Connected Component (LCC) decreases sharply. Since it is closer to complex attack patterns such as continuous penetration and coordinated strikes in real scenarios, this paper adopts the successive node attack experiment as the core evaluation paradigm.

Generally, the damage degree of a network after being attacked can be measured by the degradation of its connectivity. Specifically, a node is regarded as failed if it is removed or disconnected from the LCC. The global failure ratio θ is defined as the proportion of the network that is no longer connected, which is mathematically expressed as:

(15) $$\theta =1-\frac{\left|LCC\right|}{|LCC_{0}|}$$

where |LCC| denotes the size of the largest connected component after the attack, and $|LCC_{0}|$ is the size of the largest connected component of the initial network. A higher failure ratio indicates a smaller remaining LCC, signifying a better damage effect, and demonstrates that the adopted importance metric can identify critical nodes more effectively.

This study conducts network attack simulation experiments on six publicly available datasets to systematically compare the performance of COWMF and other node importance metrics under deliberate node removal. The attack strategy sequentially removes nodes based on the importance rankings generated by each metric, while monitoring the evolution of the network’s largest connected component. Through statistical analysis of the simulation results, this study evaluates the capability of different metrics to identify critical nodes that influence network structural stability, providing a reference for resilience modeling and protection strategies in complex networks.

**Figure 6:**
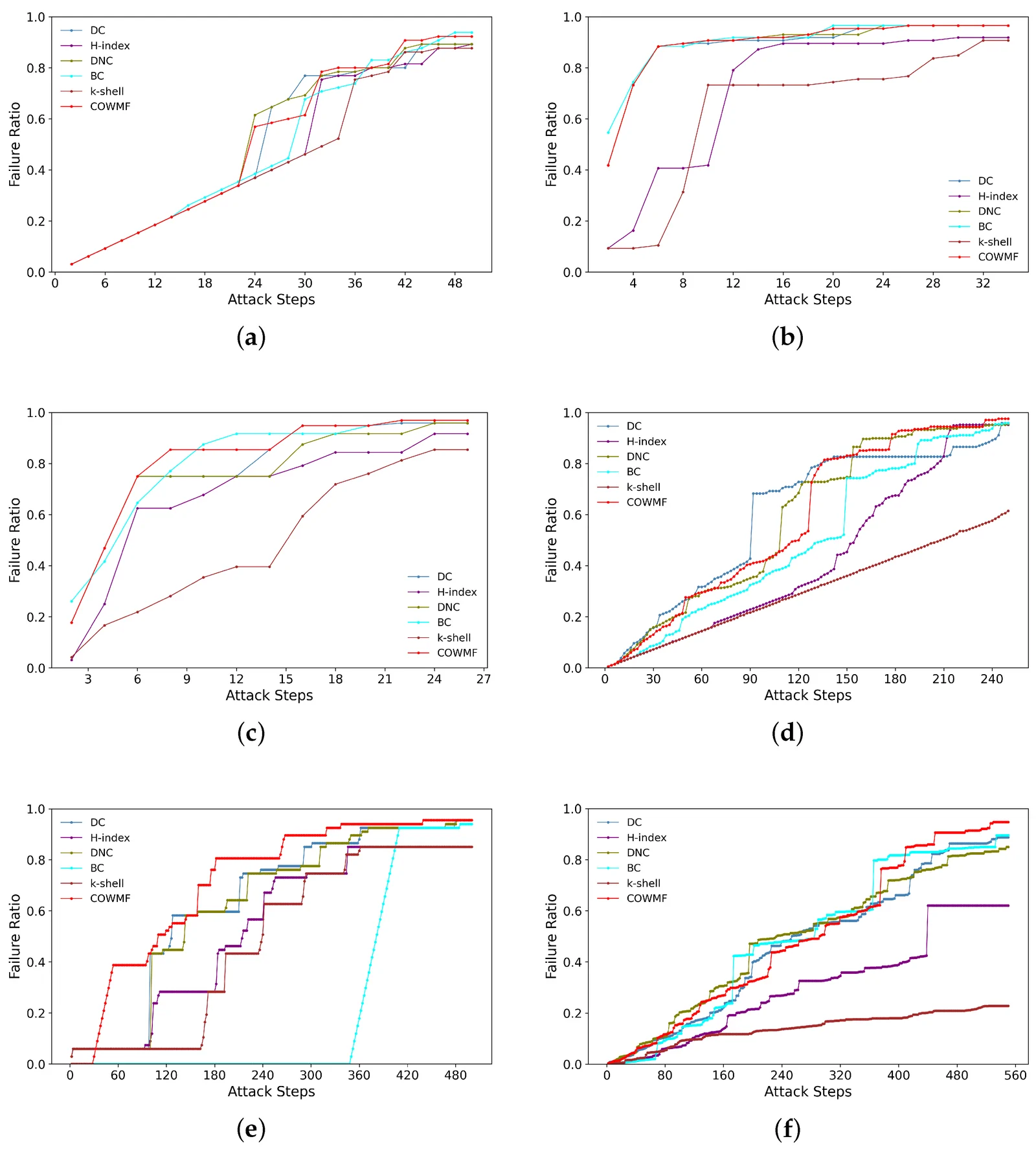
Comparison of network robustness under targeted node removal when top-ranked nodes identified by the COWMF importance indicator and classical importance metrics are sequentially removed in six real-world networks. (**a**) BrainCat; (**b**) sandi-auths; (**c**) retweet; (**d**) Anaheim_traffic; (**e**) SC-TS; (**f**) US-Grid. The horizontal axis denotes the number of removed nodes, and the vertical axis shows the failure ratio. A higher curve indicates a more effective attack strategy.

**Figure 7:**
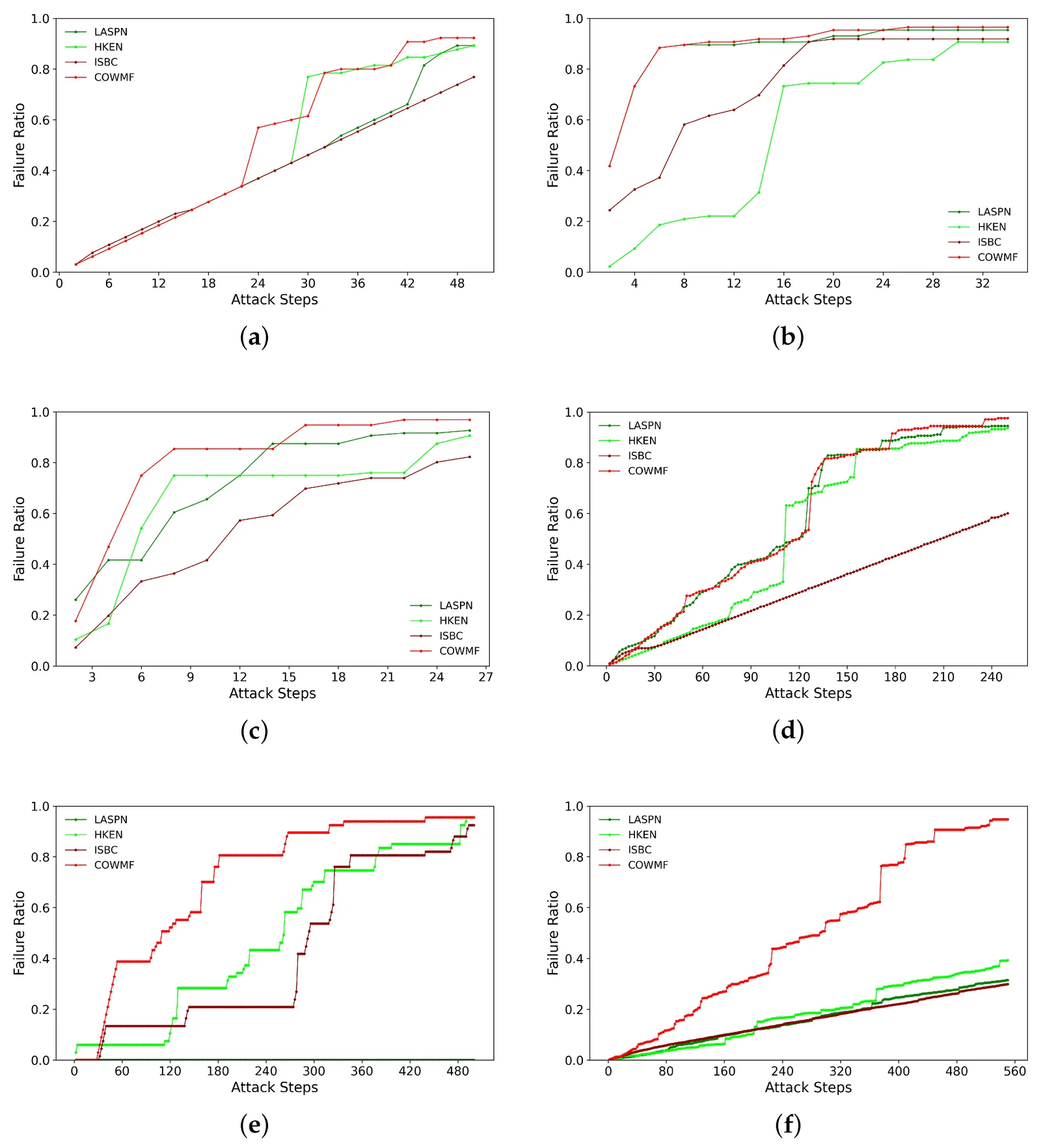
Comparison of network robustness under targeted node removal when top-ranked nodes identified by the COWMF importance indicator and recently proposed importance metrics are sequentially removed in six real-world networks. (**a**) BrainCat; (**b**) sandi-auths; (**c**) retweet; (**d**) Anaheim_traffic; (**e**) SC-TS; (**f**) US-Grid. The horizontal axis denotes the number of removed nodes, and the vertical axis shows the failure ratio. A higher curve indicates a more effective attack strategy.

Specifically, this paper employs different node importance metrics to rank network nodes and subsequently implements deliberate attacks based on these rankings to quantitatively analyze the extent of network failure during the node removal process. The experimental results, as shown in Figure 6 and Figure 7, present the network failure curves of six real-world networks, including biological, social, transportation, and power systems, under various attack strategies. The horizontal axis denotes the number of removed nodes, while the vertical axis represents the network failure ratio; a higher value indicates a greater degree of network damage. As shown in the figures, the proposed COWMF method demonstrates a generally effective network disruption capability across all tested networks.

Figure 6 compares COWMF with classical node importance metrics, including DC, H-index, DNC, BC, and k-shell, while Figure 7 compares COWMF with recently proposed metrics, namely LASPN, HKEN, and ISBC. For a more quantitative comparison, we calculated the area under the curve (AUC) of the damage curves for each method across the six networks, as shown in Table 12. The detailed rankings and average ranks are presented in Table 13. In the table, Avg Rank denotes the average rank, where a smaller value indicates a higher position. In cases of identical average ranks, the final ordering is determined by the standard deviation of the ranks across networks; a smaller standard deviation indicates higher stability and results in a higher ranking. Ties are allowed when calculating individual rankings within each network.

As shown in the figures and table, COWMF achieves the relatively favorable average rank (1.67) and demonstrates stable performance. It ranks first in the retweet, SC-TS, and US-Grid networks, second in BrainCat and sandi-auths, and third in Anaheim_traffic, performing reasonably well across most networks. In contrast, the performance of other methods is varies across different networks. For instance, BC ranks first in sandi-auths but ranks eighth in SC-TS. Similarly, LASPN performs moderately in Anaheim_traffic but shows lower performance in BrainCat and SC-TS.

In summary, existing importance metrics often rely on specific topological features and lack robustness in cross-network scenarios. In contrast, COWMF demonstrates accelerated connectivity collapse across the six tested real-world networks and maintains relatively higher network disruption performance. These results verify the reliability and broad applicability of COWMF as a general method for critical node identification, contributing to improved accuracy in network damage assessment.

### 4.2. Node Ranking Monotonicity Analysis

Node Ranking Monotonicity (NRM) is used to evaluate the discriminative power of an importance metric in distinguishing node importance within a network [44]. It is defined as:

$$\mathrm{NRM}=1-\frac{\sum_{s}N_{s}\left(N_{s}-1\right)}{N(N-1)}$$

where *s* iterates over all distinct importance scores, $N_{s}$ denotes the number of nodes assigned the same score, and *N* is the total number of nodes in the network. The NRM value ranges within [0,1]. A value closer to 1 indicates that the metric has stronger discriminative capability and yields a more refined ranking of nodes. Conversely, a value closer to 0 implies that a large number of nodes are assigned identical importance scores, reflecting limited resolution of the metric.

We compute the NRM values of various centrality metrics on the six real-world networks, with results presented in Table 14. As shown, COWMF achieves NRM values close to 1 across all six networks, significantly outperforming all baseline metrics. This demonstrates that COWMF exhibits superior ranking monotonicity and possesses the capability to assign unique importance scores to almost all nodes, thereby avoiding the ambiguity caused by tied values.

## 5. Conclusions and Future Work

To address the limitations of existing importance metrics, which often fail to jointly capture local connectivity, semi-local spreading capacity, and global structural roles, and suffer from score concentration and insufficient discriminative power, this paper proposes COWMF, an improved CRITIC-based framework for critical node identification. The framework first constructs GAT-VGL, taking four low-complexity topological metrics, DC, H-index, DNC, and k-shell, as input. Through a learnable attention mechanism, GAT-VGL adaptively aggregates multi-hop neighborhood information and explicitly models global context using a virtual node, thereby generating a global influence score with high discriminative power and high computational efficiency. This score is then integrated with DC and DNC within the improved CRITIC-based objective weighting fusion scheme, enabling adaptive synergy among local connectivity strength, semi-local spreading capacity, and global structural information. Experiments on six real-world systems, including brain networks, collaboration networks, retweet networks, transportation road networks, biological networks, and power networks, demonstrate that COWMF ranks among the competitive methods in both network attack simulation and SIR spreading simulation, two complementary evaluation paradigms. Moreover, the importance scores produced by COWMF exhibit high node ranking monotonicity across all networks, with high discriminative power. In contrast to existing methods, which often show inconsistent performance between disruption and spreading tasks or are sensitive to specific network structures, COWMF achieves high performance across scenarios and networks without task-specific parameter tuning, showcasing satisfactory robustness, generalization ability, and practical utility.

This study validates the effectiveness of COWMF on single-layer, undirected, unweighted networks. However, opportunities remain for extension to more realistic complex network settings. For instance, in directed networks, node influence is direction-dependent; in weighted networks, edge strength significantly affects information flow; and in multilayer networks, interlayer coupling relationships must be coordinated. The current method has not yet been adapted to these characteristics. Furthermore, while the equal-frequency binning strategy ensures robustness, we acknowledge that it may lead to high entropy values approaching unity in specific topologies, suggesting a need to explore adaptive binning or alternative dispersion measures in future iterations. Additionally, although we have reported the standard deviation across independent SIR simulations to reflect performance variability, future work will further incorporate paired statistical significance tests to rigorously quantify the stability of the proposed method. Future work will generally focus on three dimensions, namely directionality awareness, weight sensitivity, and multilayer integration, to redesign the attribute set and fusion mechanism, thereby enhancing the applicability and robustness of the approach in real-world complex systems.

## Figures and Tables

**Table 1:** Computational time comparison between GAT-VGL inference and BC on synthetic networks.

Network Model	Nodes	Edges	GAT-VGL Time (s)	BC Time (s)
BA	5000	19,984	1.9937	43.4349
BA	10,000	39,984	4.6060	194.2556
WS	5000	15,000	0.2673	41.8025
WS	10,000	30,000	0.6283	179.5794
ER	5000	24,936	1.1892	49.4955
ER	10,000	100,057	11.6484	311.0865

**Table 2:** Statistical properties of real networks.

Dataset	Network Type	V	E	Dmax	KSmax
BrainCat	Brain Network	65	730	45	16
sandi-auths	Collaboration Networks	86	124	12	4
retweet	Retweet Networks	96	117	17	3
Anaheim_traffic	Transportation Road Network	416	634	7	3
SC-TS	Biological Networks	636	3959	66	66
US-Grid	Power Networks	4941	6594	19	5

**Table 3:** SIR Spreading Simulation Results (Networks 1–3). Parameters: infection rate β calculated by Equation (13), recovery rate γ=0.5.

Metric	BrainCat				Sandi-Auths				Retweet			
	**Final Size**		**AUC**		**Final Size**		**AUC**		**Final Size**		**AUC**	
	**Mean**	**Std**	**Mean**	**Std**	**Mean**	**Std**	**Mean**	**Std**	**Mean**	**Std**	**Mean**	**Std**
DC	53.12	5.14	2553.16	245.72	13.97	3.61	685.65	173.54	14.52	3.33	711.33	158.59
H-index	51.88	5.80	2485.40	275.61	9.91	2.74	487.54	129.64	11.77	3.28	578.10	156.40
DNC	53.10	5.10	2546.05	243.08	13.69	3.45	672.73	165.83	14.46	3.79	709.05	180.89
BC	53.30	6.28	2553.34	297.38	13.47	3.82	659.95	182.60	14.75	3.75	722.87	178.20
k-shell	48.80	10.20	2316.09	478.24	6.04	1.32	299.54	61.17	11.52	3.45	565.05	164.01
COWMF	53.23	4.40	2555.03	210.76	13.70	3.93	671.92	187.50	15.18	4.46	742.01	212.51
LASPN	51.46	7.94	2429.73	373.68	13.88	3.76	680.22	179.65	13.81	3.64	677.42	173.79
HKEN	52.66	6.26	2523.16	296.06	6.23	2.20	307.71	99.73	11.18	3.21	549.11	152.46
ISBC	50.78	9.03	2399.62	422.05	11.26	3.93	551.39	183.13	11.42	3.31	560.63	156.44

**Table 4:** SIR Spreading Simulation Results (Networks 4–6). Parameters: infection rate β calculated by Equation (13), recovery rate γ=0.5.

Metric	Anaheim_Traffic				SC-TS				US-Grid			
	**Final Size**		**AUC**		**Final Size**		**AUC**		**Final Size**		**AUC**	
	**Mean**	**Std**	**Mean**	**Std**	**Mean**	**Std**	**Mean**	**Std**	**Mean**	**Std**	**Mean**	**Std**
DC	42.21	5.26	2078.36	252.17	67.00	0.00	3331.04	1.15	582.67	19.59	28606.35	930.08
H-index	32.06	3.55	1584.69	169.48	67.00	0.00	3331.28	1.04	449.35	17.62	22137.53	837.00
DNC	44.47	4.98	2183.70	236.53	67.00	0.00	3331.24	1.21	537.21	19.46	26408.62	929.07
BC	35.61	4.19	1754.71	197.47	135.30	10.59	6587.06	500.35	408.45	16.32	20142.42	773.48
k-shell	28.23	2.82	1400.16	134.64	67.00	0.00	3331.30	1.18	336.12	10.80	16663.63	512.48
COWMF	42.69	5.39	2100.16	256.65	183.37	8.03	8970.16	380.86	563.93	22.07	27695.33	1057.05
LASPN	39.87	4.77	1962.18	223.99	48.26	5.04	2382.22	233.29	390.21	16.64	19281.74	793.52
HKEN	30.78	3.40	1522.63	162.67	67.00	0.00	3331.23	1.05	351.56	12.55	17420.08	598.87
ISBC	28.56	3.10	1416.96	149.24	158.66	20.23	7600.10	927.94	412.08	17.82	20342.33	851.57

**Table 5:** Ranking details, average rank, and standard deviation of selected methods based on Spreading AUC from SIR spreading simulation experiments across six real-world networks.

Rank	Method	BrainCat	Sandi-Auths	Retweet	Anaheim_TRAFFIC	SC-TS	US-Grid	Avg Rank	Std Dev
1	COWMF	1	4	1	2	1	2	1.83	1.07
2	DC	3	1	3	3	8	1	3.17	2.19
3	DNC	4	3	4	1	6	3	3.50	1.50
4	BC	2	5	2	5	3	6	3.83	1.57
5	H-index	6	7	6	6	5	4	5.67	0.94
6	LASPN	7	2	5	4	9	7	5.67	2.29
7	ISBC	8	6	8	8	2	5	6.17	2.03
8	HKEN	5	8	9	7	7	8	7.33	1.25
9	k-shell	9	9	7	9	4	9	7.83	1.77

**Table 6:** SIR Spreading Simulation Results (Networks 1–3). Parameters: infection rate β=0.05, recovery rate γ=0.5.

Metric	BrainCat				Sandi-Auths				Retweet			
	**Final Size**		**AUC**		**Final Size**		**AUC**		**Final Size**		**AUC**	
	**Mean**	**Std**	**Mean**	**Std**	**Mean**	**Std**	**Mean**	**Std**	**Mean**	**Std**	**Mean**	**Std**
DC	33.42	9.59	1590.95	445.33	9.79	2.48	481.94	119.04	10.31	2.62	507.35	125.08
H-index	32.42	9.24	1540.38	423.81	7.53	1.86	372.27	89.14	8.69	2.28	428.36	109.34
DNC	33.02	9.89	1570.39	454.24	9.64	2.73	474.25	130.28	9.90	2.66	487.29	127.80
BC	33.53	9.98	1589.16	460.90	9.04	2.49	446.15	119.30	9.75	2.43	479.81	115.57
k-shell	28.28	12.30	1331.87	559.22	5.26	0.58	262.65	27.68	7.70	2.10	380.63	99.01
COWMF	31.99	9.36	1522.29	428.93	10.03	2.79	494.31	134.91	9.90	2.70	487.11	129.62
LASPN	29.10	11.36	1356.94	507.96	9.40	2.41	463.40	115.18	9.79	2.68	482.27	128.24
HKEN	34.88	9.80	1652.68	450.06	5.40	0.72	269.38	34.80	8.08	1.75	399.72	84.37
ISBC	28.48	12.78	1326.18	564.81	7.59	1.97	375.44	94.29	8.58	2.40	422.54	113.03

**Table 7:** SIR Spreading Simulation Results (Networks 4–6). Parameters: infection rate β=0.05, recovery rate γ=0.5.

Metric	Anaheim_Traffic				SC-TS				US-Grid			
	**Final Size**		**AUC**		**Final Size**		**AUC**		**Final Size**		**AUC**	
	**Mean**	**Std**	**Mean**	**Std**	**Mean**	**Std**	**Mean**	**Std**	**Mean**	**Std**	**Mean**	**Std**
DC	32.26	3.55	1595.95	169.89	66.69	0.58	3309.28	28.65	411.42	13.70	20322.83	656.90
H-index	26.25	2.88	1304.26	138.63	66.75	0.61	3310.68	29.94	348.84	12.32	17281.46	591.56
DNC	31.90	3.16	1578.49	151.14	66.76	0.51	3311.68	25.15	391.01	11.82	19330.10	570.64
BC	27.96	3.18	1385.75	152.98	91.89	11.23	4475.48	524.94	324.76	9.50	16112.46	455.41
k-shell	24.43	1.83	1216.39	88.39	66.69	0.61	3308.61	30.24	289.99	7.38	14435.88	353.86
COWMF	31.55	4.03	1560.57	192.01	147.28	8.56	7143.37	405.80	406.27	12.98	20067.63	622.41
LASPN	29.68	3.70	1470.23	178.32	39.63	3.52	1967.08	166.76	319.19	9.28	15850.83	446.05
HKEN	25.58	2.49	1271.54	119.01	66.75	0.52	3311.82	25.84	299.68	8.84	14906.54	425.67
ISBC	24.34	1.78	1212.39	86.40	107.61	23.44	5122.16	1081.36	328.94	8.96	16322.75	431.69

**Table 8:** SIR Spreading Simulation Results (Networks 1–3). Parameters: infection rate β=0.2, recovery rate γ=0.5.

Metric	BrainCat				Sandi-Auths				Retweet			
	**Final Size**		**AUC**		**Final Size**		**AUC**		**Final Size**		**AUC**	
	**Mean**	**Std**	**Mean**	**Std**	**Mean**	**Std**	**Mean**	**Std**	**Mean**	**Std**	**Mean**	**Std**
DC	61.82	2.43	3014.35	116.74	23.27	5.61	1136.81	267.31	22.55	4.75	1102.74	227.24
H-index	60.79	2.48	2956.76	120.23	14.54	4.79	709.45	220.52	19.81	5.28	967.13	251.47
DNC	61.67	2.00	3007.97	98.27	22.97	5.64	1121.20	268.26	23.58	5.74	1151.15	273.81
BC	61.64	2.17	3003.70	105.24	20.33	5.37	994.90	256.98	23.65	5.02	1155.91	239.22
k-shell	61.60	2.07	2978.83	100.50	7.61	3.39	373.87	157.45	18.27	6.12	890.15	289.22
COWMF	62.00	1.99	3025.07	97.21	22.23	5.21	1087.34	248.76	23.90	5.20	1165.66	245.89
LASPN	60.76	2.29	2926.43	112.15	23.91	5.66	1165.62	267.57	23.86	6.12	1162.64	289.36
HKEN	61.42	2.24	2986.40	108.28	7.77	3.55	379.65	154.73	17.78	5.42	865.47	251.51
ISBC	61.75	2.89	2973.18	139.20	16.34	5.36	797.73	251.88	19.55	6.30	949.58	293.78

**Table 9:** SIR Spreading Simulation Results (Networks 4–6). Parameters: infection rate β=0.2, recovery rate γ=0.5.

Metric	Anaheim_Traffic				SC-TS				US-Grid			
	**Final Size**		**AUC**		**Final Size**		**AUC**		**Final Size**		**AUC**	
	**Mean**	**Std**	**Mean**	**Std**	**Mean**	**Std**	**Mean**	**Std**	**Mean**	**Std**	**Mean**	**Std**
DC	66.22	8.01	3231.03	375.54	67.00	0.00	3332.47	0.17	908.74	31.03	44376.16	1472.77
H-index	43.61	5.88	2136.84	275.23	67.00	0.00	3332.45	0.22	668.02	27.05	32675.23	1273.94
DNC	66.16	8.89	3228.24	419.77	67.00	0.00	3332.47	0.22	823.72	33.09	40278.97	1577.60
BC	52.86	6.82	2581.43	319.24	180.79	9.91	8841.65	469.06	599.14	26.00	29277.71	1216.20
k-shell	36.19	4.68	1782.38	219.59	67.00	0.00	3332.47	0.17	426.42	16.83	21015.23	791.35
COWMF	64.65	8.90	3157.97	418.08	216.46	9.93	10621.70	456.67	875.14	30.09	42746.18	1425.55
LASPN	60.76	8.33	2960.91	388.24	60.15	6.49	2958.49	303.20	522.16	19.97	25665.75	937.08
HKEN	41.98	6.73	2058.32	314.53	67.00	0.00	3332.49	0.10	452.49	18.62	22293.58	887.87
ISBC	37.60	4.63	1851.54	216.30	214.62	14.45	10353.54	672.76	590.20	27.33	28904.41	1272.04

**Table 10:** SIR Spreading Simulation Results (Networks 1–3). Parameters: infection rate β=0.5, recovery rate γ=0.5.

Metric	BrainCat				Sandi-Auths				Retweet			
	**Final Size**		**AUC**		**Final Size**		**AUC**		**Final Size**		**AUC**	
	**Mean**	**Std**	**Mean**	**Std**	**Mean**	**Std**	**Mean**	**Std**	**Mean**	**Std**	**Mean**	**Std**
DC	64.40	0.76	3174.22	36.92	44.22	6.57	2161.86	314.72	42.49	7.17	2076.30	341.86
H-index	64.21	0.79	3158.28	37.62	28.71	8.54	1393.53	396.93	39.87	6.81	1942.52	322.81
DNC	64.42	0.72	3175.96	34.91	44.08	6.30	2154.88	301.32	45.30	7.84	2213.61	376.35
BC	64.45	0.62	3176.41	30.30	41.01	7.76	2001.35	369.49	45.33	6.93	2216.26	331.43
k-shell	64.55	0.65	3164.20	31.95	16.11	10.03	774.85	461.48	34.51	7.56	1680.49	359.71
COWMF	64.38	0.72	3173.33	35.37	42.97	7.10	2103.41	339.71	45.43	6.06	2219.64	289.26
LASPN	64.08	0.92	3131.09	45.58	41.31	5.84	2021.22	278.25	44.16	7.01	2156.69	335.13
HKEN	64.26	0.80	3160.57	38.45	15.98	10.74	763.85	488.97	34.89	8.67	1697.96	410.14
ISBC	64.48	0.74	3154.90	35.66	33.68	9.64	1636.54	456.22	38.78	9.00	1881.86	425.69

**Table 11:** SIR Spreading Simulation Results (Networks 4–6). Parameters: infection rate β=0.5, recovery rate γ=0.5.

Metric	Anaheim_Traffic				SC-TS				US-Grid			
	**Final Size**		**AUC**		**Final Size**		**AUC**		**Final Size**		**AUC**	
	**Mean**	**Std**	**Mean**	**Std**	**Mean**	**Std**	**Mean**	**Std**	**Mean**	**Std**	**Mean**	**Std**
DC	132.06	15.59	6385.40	711.84	67.00	0.00	3332.50	0.00	1771.83	47.92	86189.85	2247.70
H-index	90.31	16.16	4346.06	735.24	67.00	0.00	3332.50	0.00	1317.76	59.66	63861.20	2741.97
DNC	139.70	16.84	6751.89	770.59	67.00	0.00	3332.50	0.00	1619.80	47.92	78717.73	2230.52
BC	110.08	15.36	5310.34	703.49	221.97	6.78	10939.97	325.37	1253.90	59.18	60456.13	2717.69
k-shell	65.26	14.91	3144.66	658.52	67.00	0.00	3332.50	0.00	690.56	43.11	33683.65	1950.97
COWMF	137.99	16.26	6651.55	746.23	248.41	11.30	12237.68	521.43	1717.56	55.18	83521.45	2587.03
LASPN	130.12	19.33	6260.29	887.05	77.41	6.66	3816.55	318.22	949.68	58.45	46056.93	2628.75
HKEN	86.35	17.74	4144.68	794.86	67.00	0.00	3332.50	0.00	758.47	50.88	36897.11	2266.58
ISBC	73.20	15.56	3520.52	700.61	285.58	12.38	13931.95	568.02	1197.84	64.47	57729.76	2914.70

**Table 12:** AUC of Damage Curves Across Different Networks under Simulated Attacks.

Network	DC	H-Index	DNC	BC	k-Shell	COWMF	LASPN	HKEN	ISBC
BrainCat	24.6462	22.8000	25.1692	23.7692	21.8308	25.0154	20.3692	23.7231	19.3846
sandi-auths	28.8488	23.3837	28.9419	29.2093	20.5814	29.0116	28.6744	17.9767	23.9302
retweet	19.3021	16.7812	18.7396	19.7500	12.0000	19.9792	17.6042	16.2188	13.2500
Anaheim_traffic	149.8582	107.1635	146.6707	125.7043	75.2356	145.9279	145.3293	131.6154	75.6250
SC-TS	318.4776	265.8358	313.1642	112.1940	241.1791	357.3731	0.0000	240.7164	209.7015
US-Grid	259.9662	166.7729	269.8755	271.2759	77.3101	273.4177	88.4647	100.6877	86.6626

**Table 13:** Ranking details, average rank, and standard deviation of methods based on AUC of damage curves under simulated attack experiments across six real-world networks.

Rank	Method	BrainCat	Sandi-Auths	Retweet	Anaheim_Traffic	SC-TS	US-Grid	Avg Rank	Std Dev
1	COWMF	2	2	1	3	1	1	1.67	0.75
2	DNC	1	3	4	2	3	3	2.67	0.94
3	DC	3	4	3	1	2	4	2.83	1.07
4	BC	4	1	2	6	8	2	3.83	2.48
5	H-index	6	7	6	7	4	5	5.83	1.07
6	HKEN	5	9	7	5	6	6	6.33	1.37
7	LASPN	8	5	5	4	9	7	6.33	1.80
8	ISBC	9	6	8	8	7	8	7.67	0.94
9	k-shell	7	8	9	9	5	9	7.83	1.46

**Table 14:** Monotonicity scores of node importance metrics across six real-world networks.

Network	DC	Hindex	DNC	BC	k-shell	LASPN	HKEN	ISBC	COWMF
BrainCat	0.9759	0.9466	0.9980	0.9985	0.6548	1.0000	1.0000	0.0908	1.0000
sandi-auths	0.7915	0.6952	0.9928	0.6322	0.6432	0.9945	0.9942	0.3767	1.0000
retweet	0.6594	0.5390	0.9899	0.6521	0.4885	0.9910	0.9907	0.5438	0.9997
Anaheim_traffic	0.7088	0.5743	0.9898	0.9987	0.1319	0.9992	0.9979	0.0475	1.0000
SC-TS	0.9022	0.8811	0.9739	0.7900	0.8728	0.9758	0.9746	0.0861	0.9828
US-Grid	0.7698	0.6269	0.9970	0.9117	0.4959	0.9999	0.9994	0.3387	0.9999

**Figure A1:**
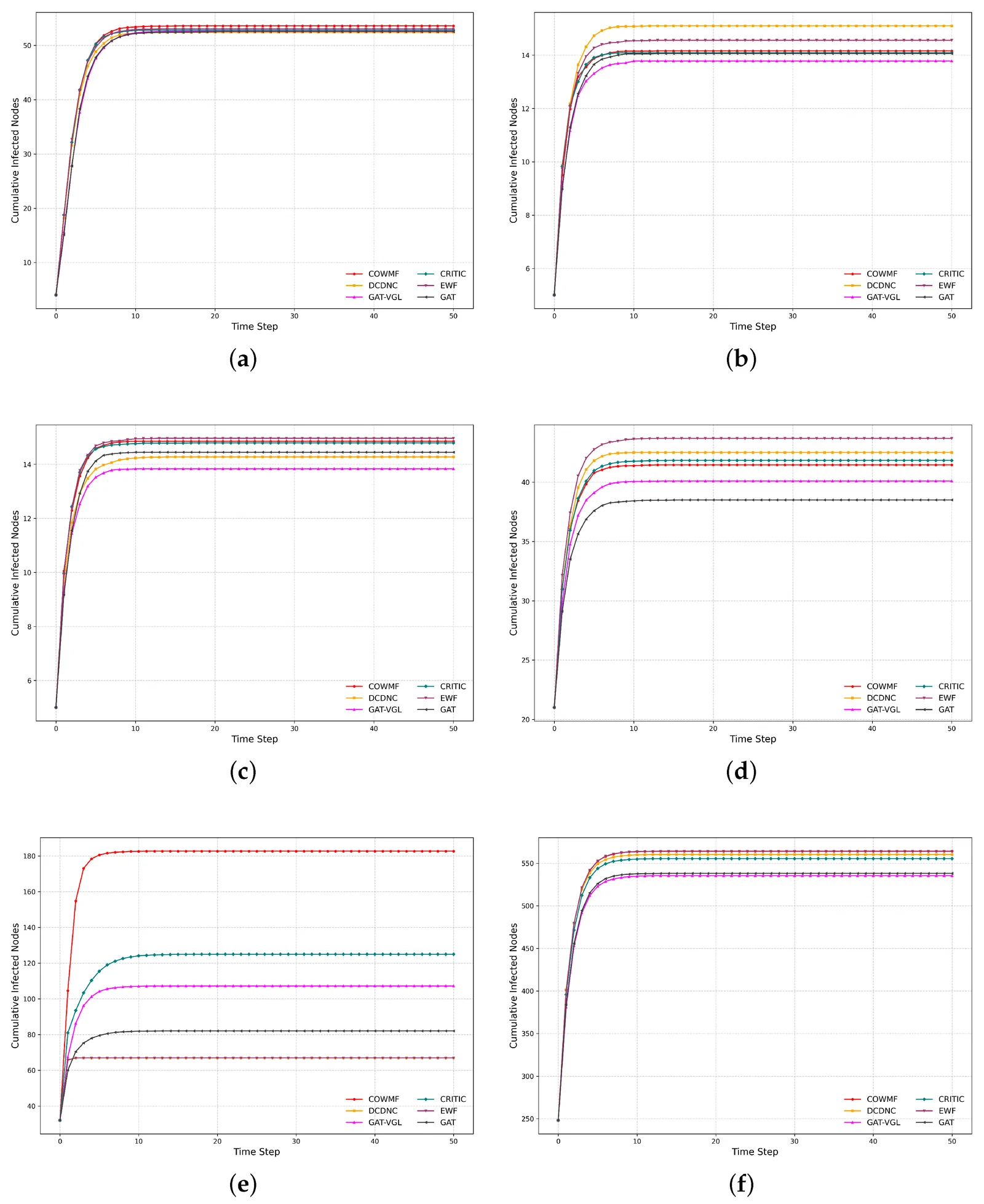
Comparison of SIR spreading dynamics when top-ranked nodes identified by the COWMF, DCDNC, GAT-VGL, GAT, CRITIC, EWF and COWMFGAT importance metrics are used as initial infection seeds across six real-world networks. (**a**) BrainCat; (**b**) sandi-auths; (**c**) retweet; (**d**) Anaheim_traffic; (**e**) SC-TS; (**f**) US-Grid. The horizontal axis denotes the time step, and the vertical axis shows the average cumulative number of infected nodes over 100 independent simulations.

**Figure A2:**
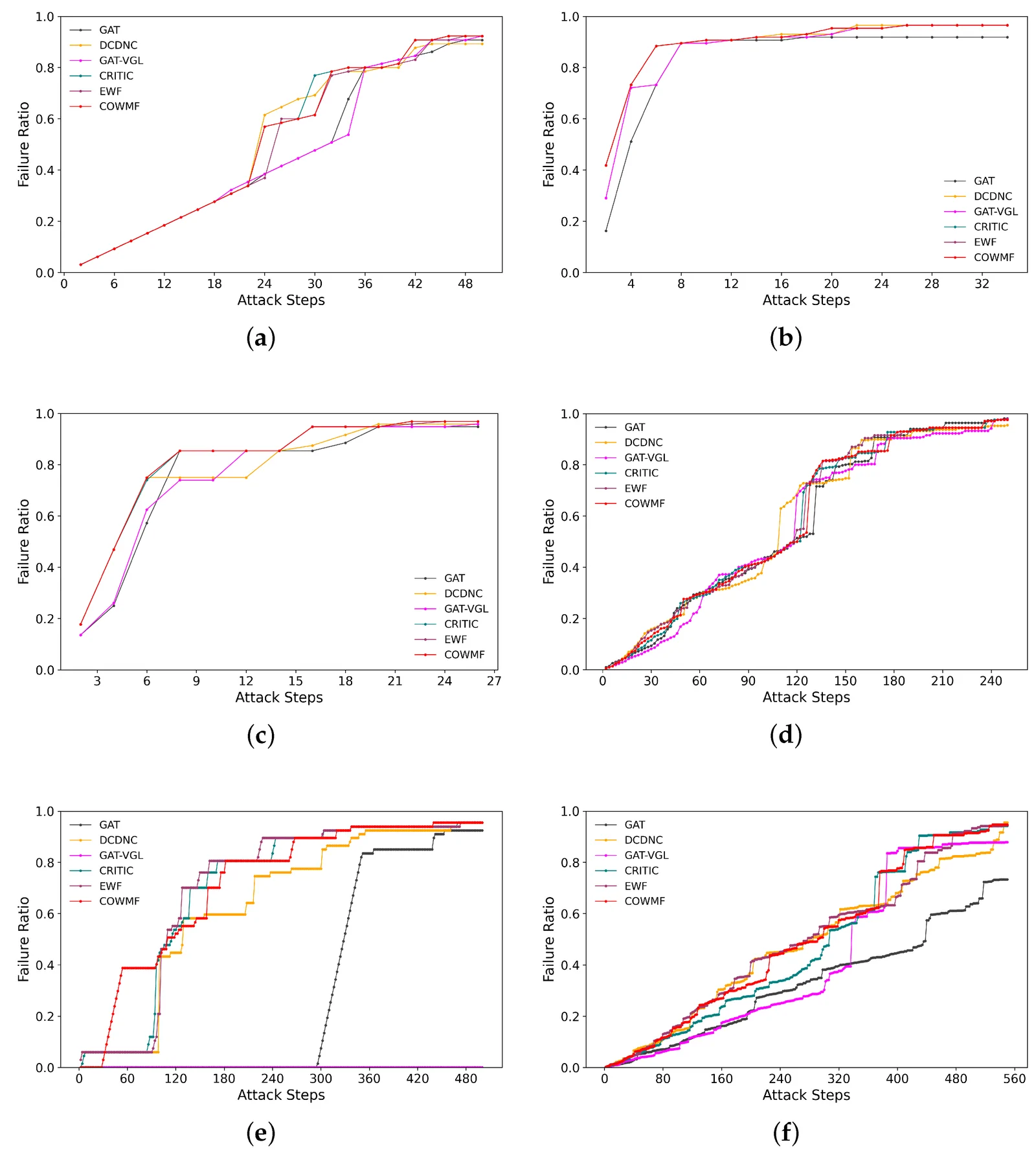
Comparison of network robustness under targeted node removal when top-ranked nodes identified by the COWMF, DCDNC, GAT-VGL, GAT, CRITIC, EWF and COWMFGAT importance metrics are sequentially removed in six real-world networks. (**a**) BrainCat; (**b**) sandi-auths; (**c**) retweet; (**d**) Anaheim_traffic; (**e**) SC-TS; (**f**) US-Grid. The horizontal axis denotes the number of removed nodes, and the vertical axis shows the failure ratio. A higher curve indicates a more effective attack strategy.

## Data Availability

The datasets used in this study are sourced from the Network Repository and ICON. The specific dataset links are as follows: https://networkrepository.com/bn-cat-mixed-species-brain-1.php (BrainCat, accessed on 10 June 2026), https://networkrepository.com/ca-sandi-auths.php (sandi-auths, accessed on 10 June 2026), https://networkrepository.com/rt-retweet.php (retweet, accessed on 10 June 2026), https://icon.colorado.edu/networks (Anaheim_traffic, accessed on 10 June 2026), https://networkrepository.com/bio-SC-TS.php (SC-TS, accessed on 10 June 2026), and https://networkrepository.com/power-US-Grid.php (US-Grid, accessed on 10 June 2026).

## Appendix A. Experimental Setup and Hyperparameter Details

This appendix provides supplementary details on the experimental configuration, including synthetic graph generation parameters, hyperparameter settings, and the detailed list of real-world networks used for fine-tuning.

### Appendix A.1. Synthetic Graph Generation Parameters

To ensure diversity in topological structures, we generate synthetic graphs using three classical random graph models: Barabási–Albert (BA), Watts–Strogatz (WS), and Erdős–Rényi (ER). The key parameters for each generator are detailed in Table A1. The number of nodes for each generated graph is uniformly sampled within the range of [50,250] to cover networks of varying scales.

**Table A1:** Key Parameters for Synthetic Graph Generators.

Network Model	Node Count (n)	Key Parameters	Description
BA (Barabási–Albert)	50∼250	m: Random integer [2, 5] (and m ≤ n/2)	m is the number of edges to attach from a new node to existing nodes.
WS (Watts–Strogatz)	50∼250	k: Randomly chosen from {2, 4, 6}; p: Random float [0.1, 0.5]	k is the number of nearest neighbors to connect, p is the rewiring probability.
ER (Erdős–Rényi)	50∼250	p: Random float [0.05, 0.2]	Uses the G(n, p) model, where p is the probability of edge creation between any two nodes.

### Appendix A.2. Hyperparameter Configuration

The hyperparameter settings for both the pre-training and fine-tuning stages are summarized in Table A2. We adopt the Adam optimizer for both stages. To mitigate catastrophic forgetting during fine-tuning, the learning rate is reduced to one-tenth of the pre-training value.

**Table A2:** Hyperparameter Configuration for Pre-training and Fine-tuning.

Parameter	Stage 1: Synthetic Graph Pre-Training	Stage 2: Real-World Graph Fine-Tuning
Optimizer	Adam	Adam
Learning Rate (LR)	$1\times 10^{-3}$	$1\times 10^{-4}$
Batch Size	10	10
Random Seed	42	42
MLP Dimensions	[64 → 32 → 1]	[64 → 32 → 1]
Attention Head Aggregation	Concat	Concat
Validation Process	Hold-out Validation Set (10%)	Independent Test Set (20%)
Model Selection Rule	Save the model with the highest Kendall Tau	Save the final model

### Appendix A.3. Real-World Networks for Fine-Tuning

The fine-tuning stage is conducted on a total of 35 real-world networks collected from various domains (e.g., bioinformatics, social networks, infrastructure). Following an 80%/20% split, these networks are divided into a training set of 28 networks and a test set of 7 networks. The detailed list is presented in Table A3.

**Table A3:** List of 35 Real-world Networks for Fine-tuning (28 Training/7 Test).

Training Set (28 Networks)		Test Set (7 Networks)
ENZYMES_g103	chesapeake	ENZYMES_g112
ENZYMES_g113	ia-crime-moreno	ENZYMES_g118
aves-barn-swallow-non-physical	ia-infect-dublin	ENZYMES_g123
aves-geese-female-foraging	ia-infect-hyper	aves-geese-male-foraging
aves-hens-pecking-order	power-494-bus	aves-weaver-social
aves-sparrow-social	power-662-bus	aves-weaver-social-08
aves-sparrow-social-2009	road-euroroad	bio-CE-LC
aves-sparrow-social-2010	road-minnesota	ia-enron-only
aves-sparrowlyon-flock-season3	soc-dolphins	
aves-thornbill-farine	soc-highschool-moreno	
aves-weaver-social-16	soc-karate	
bio-DM-LC	soc-physicians	
bio-celegans	soc-student-coop	
bio-diseasome	soc-tribes	

### Appendix A.4. Sensitivity Analysis of Quantile Bin Number (B) and Correlation Metric

This section presents the sensitivity analysis results for the proposed weighting mechanism under different configurations. Two factors are examined: (1) the choice of correlation measure (Pearson vs. Spearman) for computing inter-metric conflict, and (2) the number of quantile bins *B* used in the entropy calculation. The analysis is conducted on two representative datasets, Anaheim_traffic and retweet, with B∈{10,20,30,50}.

**Table A4:** Comparison of Pearson and Spearman Correlation on Anaheim_traffic.

Network Dataset: Anaheim_Traffic.									
**Strategy**	**Information Entropy (H)**			**Conflict (C)**			**Weight (W)**		
	**DC**	**DNC**	**GAT-VGL**	**DC**	**DNC**	**GAT-VGL**	**DC**	**DNC**	**GAT-VGL**
Pearson	0.8474	0.9887	0.9999	0.1621	0.1788	0.2630	0.2380	0.3063	0.4557
Spearman	0.8474	0.9887	0.9999	0.1560	0.1650	0.2220	0.2556	0.3154	0.4290

**Table A5:** Comparison of Pearson and Spearman Correlation on retweet.

Network Dataset: Retweet									
**Strategy**	**Information Entropy (H)**			**Conflict (C)**			**Weight (W)**		
	**DC**	**DNC**	**GAT-VGL**	**DC**	**DNC**	**GAT-VGL**	**DC**	**DNC**	**GAT-VGL**
Pearson	0.8503	0.9782	0.9978	0.2641	0.4001	0.5739	0.1889	0.3293	0.4818
Spearman	0.8503	0.9782	0.9978	0.3468	0.5277	0.6648	0.2000	0.3501	0.4499

**Table A6:** Sensitivity Analysis of CRITIC Weights to the Number of Equal-Frequency Bins (*B*) on Anaheim_traffic.

Network Dataset: Anaheim_Traffic.									
**Bin**	**Information Entropy (H)**			**Conflict (C)**			**Weight (W**)		
	**DC**	**DNC**	**GAT-VGL**	**DC**	**DNC**	**GAT-VGL**	**DC**	**DNC**	**GAT-VGL**
10	0.8474	0.9967	0.9999	0.1621	0.1788	0.2630	0.2374	0.3081	0.4545
20	0.8474	0.9907	0.9999	0.1621	0.1788	0.2630	0.2378	0.3068	0.4554
30	0.8474	0.9887	0.9999	0.1621	0.1788	0.2630	0.2380	0.3063	0.4557
50	0.8474	0.9808	0.9996	0.1621	0.1788	0.2630	0.2386	0.3047	0.4567

**Table A7:** Sensitivity Analysis of CRITIC Weights to the Number of Equal-Frequency Bins (*B*) on retweet.

Network Dataset: Retweet									
**Bin**	**Information Entropy (H)**			**Conflict (C)**			**Weight (W)**		
	**DC**	**DNC**	**GAT-VGL**	**DC**	**DNC**	**GAT-VGL**	**DC**	**DNC**	**GAT-VGL**
10	0.8503	0.9936	0.9994	0.2611	0.4001	0.5739	0.1878	0.3325	0.4797
20	0.8503	0.9861	0.9988	0.2611	0.4001	0.5739	0.1883	0.3309	0.4808
30	0.8503	0.9782	0.9978	0.2611	0.4001	0.5739	0.1889	0.3293	0.4818
50	0.8503	0.9776	0.9956	0.2611	0.4001	0.5739	0.1892	0.3295	0.4813

### Appendix A.5. Ablation Study and Extended Comparative Experiments

This section presents additional comparative experiments involving DCDNC, GAT-VGL, GAT, CRITIC, EWF, and COWMFGAT. Notably, DCDNC is a combined centrality measure that integrates DC and DNC scores using the same fusion strategy as COWMF. GAT serves as a control experiment with the same architecture as GAT-VGL but without virtual nodes. CRITIC refers to the traditional CRITIC method, while EWF denotes equal-weight fusion; these two are included as control experiments to compare against the improved CRITIC strategy in COWMF, utilizing the same attributes but with different fusion strategies. Furthermore, COWMFGAT employs the same fusion strategy as COWMF but replaces GAT-VGL with GAT.

#### Appendix A.5.1. SIR Spreading Simulation (Additional Results)

We compare COWMF with DCDNC, GAT-VGL, GAT, CRITIC, EWF, and COWMFGAT on six real-world networks. Figure A1 shows the infection curves when the top 5% nodes identified by each method are used as initial seeds. Detailed performance metrics are presented in Table A8 and Table A9, and the overall rankings are summarized in Table A10. As shown in Table A10, COWMF achieves the favorable average rank (3.50) among all 15 methods, demonstrating good and stable performance in SIR spreading simulation. In the table, Avg Rank denotes the average rank, and Rank is determined based on the average rank, where a smaller average rank indicates a higher position. In cases of identical average ranks, the final ordering is determined by the standard deviation of the ranks across networks; a smaller standard deviation indicates higher stability and results in a higher ranking. When calculating the individual rankings within each network, ties are allowed. From Table A10, GAT-VGL and GAT obtain nearly identical average ranks (8.33 each), indicating similar performance of the two GNN variants. However, COWMF (which incorporates GAT-VGL) achieves a better average rank of 3.50 compared to COWMFGAT (6.00), suggesting that GAT-VGL contributes more effectively to the fusion framework than GAT. This highlights the advantage of GAT-VGL in capturing global information through virtual nodes.

**Table A8:** SIR Spreading Simulation Results (Networks 1–3) with Extended Methods. Parameters: infection rate β calculated by Equation (13), recovery rate γ=0.5.

Metric	BrainCat				Sandi-Auths				Retweet			
	**Final Size**		**AUC**		**Final Size**		**AUC**		**Final Size**		**AUC**	
	**Mean**	**Std**	**Mean**	**Std**	**Mean**	**Std**	**Mean**	**Std**	**Mean**	**Std**	**Mean**	**Std**
DC	53.12	5.14	2553.16	245.72	13.97	3.61	685.65	173.54	14.52	3.33	711.33	158.59
H-index	51.88	5.80	2485.40	275.61	9.91	2.74	487.54	129.64	11.77	3.28	578.10	156.40
DNC	53.10	5.10	2546.05	243.08	13.69	3.45	672.73	165.83	14.46	3.79	709.05	180.89
BC	53.30	6.28	2553.34	297.38	13.47	3.82	659.95	182.60	14.75	3.75	722.87	178.20
k-shell	48.80	10.20	2316.09	478.24	6.04	1.32	299.54	61.17	11.52	3.45	565.05	164.01
COWMF	53.23	4.40	2555.03	210.76	13.70	3.93	671.92	187.50	15.18	4.46	742.01	212.51
LASPN	51.46	7.94	2429.73	373.68	13.88	3.76	680.22	179.65	13.81	3.64	677.42	173.79
HKEN	52.66	6.26	2523.16	296.06	6.23	2.20	307.71	99.73	11.18	3.21	549.11	152.46
ISBC	50.78	9.03	2399.62	422.05	11.26	3.93	551.39	183.13	11.42	3.31	560.63	156.44
DCDNC	53.71	5.32	2583.62	253.79	14.57	3.79	713.90	180.62	14.30	3.95	700.86	189.32
GAT-VGL	52.71	6.09	2520.20	286.13	13.50	3.91	662.59	187.09	13.47	3.64	660.30	173.28
CRITIC	55.24	4.06	2645.12	191.83	13.99	3.98	687.43	191.73	14.87	4.31	728.70	205.49
EWF	52.35	5.25	2517.07	246.74	14.29	3.51	700.01	167.03	14.89	4.28	729.37	204.93
GAT	52.38	6.46	2496.71	304.74	14.13	3.85	691.65	183.53	13.52	3.94	663.18	188.83
COWMFGAT	52.12	6.22	2506.00	297.25	13.25	3.30	651.29	158.26	14.90	4.23	730.17	202.26

**Table A9:** SIR Spreading Simulation Results (Networks 4–6) with Extended Methods. Parameters: infection rate β calculated by Equation (13), recovery rate γ=0.5.

Metric	Anaheim_Traffic				SC-TS				US-Grid			
	**Final Size**		**AUC**		**Final Size**		**AUC**		**Final Size**		**AUC**	
	**Mean**	**Std**	**Mean**	**Std**	**Mean**	**Std**	**Mean**	**Std**	**Mean**	**Std**	**Mean**	**Std**
DC	42.21	5.26	2078.36	252.17	67.00	0.00	3331.04	1.15	582.67	19.59	28606.35	930.08
H-index	32.06	3.55	1584.69	169.48	67.00	0.00	3331.28	1.04	449.35	17.62	22137.53	837.00
DNC	44.47	4.98	2183.70	236.53	67.00	0.00	3331.24	1.21	537.21	19.46	26408.62	929.07
BC	35.61	4.19	1754.71	197.47	135.30	10.59	6587.06	500.35	408.45	16.32	20142.42	773.48
k-shell	28.23	2.82	1400.16	134.64	67.00	0.00	3331.30	1.18	336.12	10.80	16663.63	512.48
COWMF	42.69	5.39	2100.16	256.65	183.37	8.03	8970.16	380.86	563.93	22.07	27695.33	1057.05
LASPN	39.87	4.77	1962.18	223.99	48.26	5.04	2382.22	233.29	390.21	16.64	19281.74	793.52
HKEN	30.78	3.40	1522.63	162.67	67.00	0.00	3331.23	1.05	351.56	12.55	17420.08	598.87
ISBC	28.56	3.10	1416.96	149.24	158.66	20.23	7600.10	927.94	412.08	17.82	20342.33	851.57
DCDNC	42.74	5.77	2101.62	275.85	67.00	0.00	3331.23	1.24	559.80	18.92	27501.54	902.61
GAT-VGL	39.44	5.28	1942.10	252.28	106.12	9.99	5187.87	470.24	537.50	22.26	26389.63	1051.68
CRITIC	41.63	5.17	2048.16	245.91	126.15	16.51	6118.80	762.87	554.66	22.25	27245.30	1070.23
EWF	43.21	5.49	2125.55	261.98	67.00	0.00	3331.28	1.11	565.72	18.80	27794.08	900.00
GAT	38.32	4.64	1887.70	221.67	81.71	7.90	4010.41	366.93	537.26	17.80	26386.31	848.85
COWMFGAT	42.82	5.87	2104.85	279.42	95.14	12.73	4641.52	579.65	563.18	23.48	27661.79	1119.50

**Table A10:** Ranking details, average rank, and standard deviation of all evaluated methods based on Spreading AUC from SIR spreading simulation experiments across six real-world networks (including DCDNC, GAT-VGL, CRITIC, EWF, GAT, and COWMFGAT).

Rank	Method	BrainCat	Sandi-Auths	Retweet	Anaheim_Traffic	SC-TS	US-Grid	Avg Rank	Std Dev
1	COWMF	3	8	1	5	1	3	3.50	2.36
2	CRITIC	1	4	4	7	4	6	4.33	1.97
3	EWF	9	2	3	2	9	2	4.50	3.18
4	DCDNC	2	1	8	4	12	5	5.33	3.57
5	COWMFGAT	10	11	2	3	6	4	6.00	3.37
6	DC	5	5	6	6	14	1	6.17	3.73
7	DNC	6	7	7	1	11	7	6.50	2.87
8	BC	4	10	5	11	3	12	7.50	3.55
9	GAT-VGL	8	9	11	9	5	8	8.33	1.80
10	GAT	11	3	10	10	7	9	8.33	2.69
11	LASPN	13	6	9	8	15	13	10.67	3.14
12	ISBC	14	12	14	14	2	11	11.17	4.22
13	H-index	12	13	12	12	9	10	11.33	1.37
14	HKEN	7	14	15	13	12	14	12.50	2.69
15	k-shell	15	15	13	15	8	15	13.50	2.57

#### Appendix A.5.2. Simulated Attack Experiment (Additional Results)

We further compare COWMF with DCDNC, GAT-VGL, GAT, CRITIC, EWF, and COWMFGAT under simulated targeted attack scenarios. Figure A2 shows the attack comparison curves. Detailed AUC values are presented in Table A11 and Table A12, and the overall rankings are summarized in Table A13. As shown in Table A13, COWMF achieves the favorable average rank (2.67) among all 15 methods, demonstrating good and stable performance. In the table, Avg Rank denotes the average rank, and Rank is determined based on the average rank, where a smaller average rank indicates a higher position. When calculating the individual rankings within each network, ties are allowed. For instance, in the BrainCat network, DCDNC and DNC are tied for second place, and the next method is ranked fourth. COWMFGAT’s average ranking reached seventh place. From Table A13, GAT-VGL and GAT obtain similar average ranks (10.67 and 10.33, respectively), indicating comparable performance of the two GNN variants. However, COWMF (incorporating GAT-VGL) outperforms COWMFGAT, confirming that GAT-VGL contributes more effectively to the fusion framework than GAT in the attack scenario. This further validates the advantage of our proposed GAT-VGL module with virtual node augmentation.

**Table A11:** AUC of Damage Curves Across Different Networks under Simulated Attacks (Extended Methods: Part 1).

Network	DC	H-Index	DNC	BC	k-Shell	COWMF	LASPN	COWMFGAT
BrainCat	24.6462	22.8000	25.1692	23.7692	21.8308	25.0154	20.3692	24.4923
sandi-auths	28.8488	23.3837	28.9419	29.2093	20.5814	29.0116	28.6744	28.1628
retweet	19.3021	16.7812	18.7396	19.7500	12.0000	19.9792	17.6042	19.6771
Anaheim_traffic	149.8582	107.1635	146.6707	125.7043	75.2356	145.9279	145.3293	148.5745
SC-TS	318.4776	265.8358	313.1642	112.1940	241.1791	357.3731	0.0000	325.8060
US-Grid	259.9662	166.7729	269.8755	271.2759	77.3101	273.4177	88.4647	255.2783

**Table A12:** AUC of Damage Curves Across Different Networks under Simulated Attacks (Extended Methods: Part 2).

Network	HKEN	ISBC	DCDNC	GAT-VGL	CRITIC	EWF	GAT
BrainCat	23.7231	19.3846	25.1692	22.5846	25.3231	24.4000	22.6615
sandi-auths	17.9767	23.9302	29.0116	28.4651	29.0116	29.0116	27.3140
retweet	16.2188	13.2500	19.1146	18.7188	19.9583	19.9583	18.7292
Anaheim_traffic	131.6154	75.6250	146.7284	142.2716	146.0962	147.3654	143.7019
SC-TS	240.7164	209.7015	318.3582	0.0000	349.2836	351.7313	154.2687
US-Grid	100.6877	86.6626	262.1342	229.9122	260.4212	271.1243	181.4752

**Table A13:** Ranking details and average rank of all evaluated methods based on AUC of damage curves under simulated attack experiments across six real-world networks.

Rank	Method	BrainCat	Sandi-Auths	Retweet	Anaheim_Traffic	SC-TS	US-Grid	Avg Rank
1	COWMF	4	2	1	7	1	1	2.67
2	EWF	7	2	2	3	2	3	3.17
3	CRITIC	1	2	2	6	3	6	3.33
4	DCDNC	2	2	7	4	6	5	4.33
5	DC	5	7	6	1	5	7	5.17
6	DNC	2	6	8	5	7	4	5.33
7	COWMFGAT	6	10	5	2	4	8	5.83
8	BC	8	1	4	12	13	2	6.67
9	GAT	11	11	9	9	12	10	10.33
10	GAT-VGL	12	9	10	10	14	9	10.67
11	H-index	10	13	12	13	8	11	11.17
12	LASPN	14	8	11	8	14	13	11.33
13	HKEN	9	15	13	11	10	12	11.67
14	ISBC	15	12	14	14	11	14	13.33
15	k-shell	13	14	15	15	9	15	13.50
